# Inflammatory factors and the risk of urolithiasis: a bidirectional Mendelian randomization study

**DOI:** 10.3389/fmed.2024.1432275

**Published:** 2024-07-03

**Authors:** Kunyuan Huang, Zheng Peng, Cheng Zha, Wei Li, Guanyun Deng, Xiaolong Chen, Yuting Luo, Zhiqiang Ji, Qing Wang, Kehua Jiang

**Affiliations:** ^1^Guizhou Medical University, Guiyang, China; ^2^Department of Urology, Guizhou Provincial People’s Hospital, Guiyang, China; ^3^Guizhou University of Traditional Chinese Medicine, Guiyang, China; ^4^Zunyi Medical University, Zunyi, China

**Keywords:** urolithiasis, inflammatory factors, risk, Mendelian randomization, causal inference

## Abstract

**Background:**

Urolithiasis is a prevalent condition encountered in urology. Over the past decade, its global incidence has been on an upward trajectory; paired with a high recurrence rate, this presents considerable health and economic burdens. Although inflammatory factors are pivotal in the onset and progression of urolithiasis, their causal linkages remain elusive.

**Method:**

Mendelian randomization (MR) is predicated upon genome-wide association studies (GWASs). It integrates bioinformatics analyses to reveal causal relationships between exposures and outcomes, rendering it an effective method with minimized bias. Drawing from a publicly accessible GWAS meta-analysis comprising 8,293 samples, we identified 41 genetic variations associated with inflammatory cytokines as instrumental variables. Outcome data on upper urinary tract stones, which included renal and ureteral stones (9,713 cases and 366,693 controls), and lower urinary tract stones, including bladder and urethral stones (1,398 cases and 366,693 controls), were derived from the FinnGen Consortium R9 dataset. By leveraging the bidirectional MR methodology, we aimed to decipher the causal interplay between inflammatory markers and urolithiasis.

**Results:**

Our study comprehensively elucidated the association between genetic inflammatory markers and urolithiasis via bidirectional Mendelian randomization. Post-MR analysis of the 41 genetic inflammation markers revealed that elevated levels of circulating interleukin-2 (IL-2) (OR = 0.921, 95% CI = 0.848–0.999) suggest a reduced risk for renal stone disease, while heightened stem cell growth factor beta (SCGF-β) (OR = 1.150, 95% CI = 1.009–1.310) and diminished macrophage inflammatory protein 1 beta (MIP-1β) (OR = 0.863, 95% CI = 0.779–0.956) levels suggest an augmented risk for lower urinary tract stones. Furthermore, renal stone disease appeared to elevate IL-2 (*β* = 0.145, 95% CI = 0.013–0.276) and cutaneous T cell-attracting chemokine (CTACK) (*β* = 0.145, 95% CI = 0.013–0.276) levels in the bloodstream, whereas lower urinary tract stones were linked to a surge in interleukin-5 (IL-5) (*β* = 0.142, 95% CI = 0.057–0.226), interleukin-7 (IL-7) (*β* = 0.108, 95% CI = 0.024–0.192), interleukin-8 (IL-8) (*β* = 0.127, 95% CI = 0.044–0.210), growth regulated oncogene alpha (GRO-α) (*β* = 0.086, 95% CI = 0.004–0.169), monokine induced by interferon-gamma (MIG) (*β* = 0.099, 95% CI = 0.008–0.191) and macrophage inflammatory protein 1 alpha (MIP-1α) (*β* = 0.126, 95% CI = 0.044–0.208) levels.

**Conclusion:**

These discoveries intimate the instrumental role of IL-2 in the onset and progression of upper urinary tract stones. SCGF-β and MIP-1β influence the development of lower urinary tract stones. Urolithiasis also impacts the expression of cytokines such as IL-2, CTACK, IL-5, IL-7, IL-8, GRO-α, MIG, and MIP-1α. There is a pressing need for further investigation to ascertain whether these biomarkers can be harnessed to prevent or treat urolithiasis.

## 1 Introduction

Urolithiasis, commonly known as urinary stone disease, is a globally pervasive medical issue affecting individuals across all age brackets, including children, adolescents, and adults (1, 2). Although current therapeutic interventions—encompassing pharmacotherapy, extracorporeal shock wave lithotripsy, and surgical measures—have yielded appreciable outcomes (3), the recurrence rate among patients remains notably high. Some datasets suggest that over 50% of patients experience recurrence within 5 years (4–6). Beyond economic strain, urolithiasis inflicts significant physical and psychological distress on patients. The etiology of this condition is multifaceted, involving genetic, racial, environmental, dietary, metabolic, and inflammatory factors. Despite the wealth of etiological research on renal stones, their exact mechanistic underpinnings remain enigmatic. Thus, deepening our grasp of its pathogenesis and unearthing novel therapeutic strategies are paramount.

Renal calculi, the most prevalent subtype of urolithiasis, consist predominantly (approximately 80%) of calcium oxalate and calcium phosphate. Approximately half of these are idiopathic calcium oxalate stones. Evidence suggests that Randall’s plaques play a cardinal role in the genesis of idiopathic calcium oxalate stones, with inflammation, immune cells, and mineralization modulating factors exerting significant influence. The formation of Randall’s plaques occurs at the subepithelial renal papillae, yet their exact genesis remains ambiguous. A study using quantitative PCR pinpointed a marked elevation in various inflammatory cytokines among renal stone patients, an increase likely decoupled from systemic inflammatory responses (7). Renal tissues exhibiting Randall’s plaques manifested increased immune cell infiltration and cell apoptosis when compared with normal tissues. Animal studies have underscored the interplay between macrophage polarization states and the formation of calcium oxalate crystals (CaOx) (8). This emphasizes the role of inflammation, oxidative stress, and apoptosis in forming Randall’s plaques and CaOx. While the functions of inflammation and immune cells in urinary stone disease have garnered attention, research into the associated inflammatory markers still needs to be conducted. Delving deeper into the correlation between inflammatory factors and urinary stone disease holds profound implications for the etiological study of urolithiasis.

For our study, we employed an observational research design using Mendelian randomization (MR) to dissect the causal nexus between inflammatory cytokines and the progression of urolithiasis. Diverging from conventional multivariable observational analyses, MR capitalizes on genetic variations as instrumental variables to estimate the causal impact of exposures on outcomes, offering a robust buffer against confounding and reverse causation (9). The inferences drawn from MR are consistent with those from randomized controlled trials (RCTs), enabling a reliable assessment of the causal ramifications of risk factors on health outcomes. Bidirectional Mendelian randomization analysis, in terms of bidirectionality and robustness of causal inference, allows for a more comprehensive understanding of complex biological mechanisms, reduces the influence of confounding factors, and thereby enhances the credibility of research conclusions. To gauge the causal linkage between variations in circulating levels of inflammatory markers and the risk of urolithiasis onset, we performed a bidirectional Mendelian randomization analysis using genetic variations as the instrumental variables (10). We excluded genetic variations interlinked with confounding factors, postulating that these genetic variations are associated with urolithiasis solely through exposures. Our MR analytical results can be used to evaluate the causal impacts of inflammatory factors on the risk of urolithiasis onset.

## 2 Materials and methods

### 2.1 Study design

**Figure 1:**
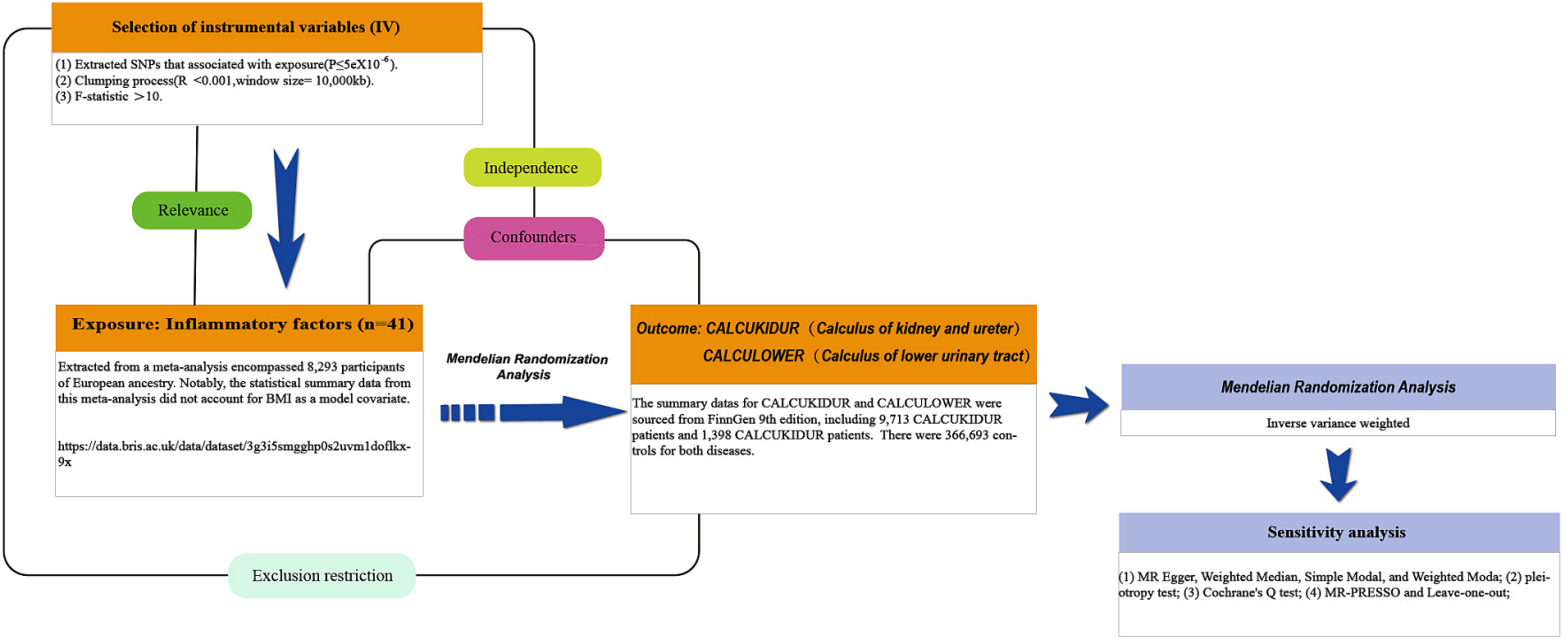
The study design schematic in this bidirectional Mendelian randomization (MR) analysis. Significant instrumental variables were selected for 41 inflammatory cytokines and urolithiasis to investigate their bidirectional causal relationship. This causal directed acyclic graph illustrates the three basic assumptions of MR analysis, namely, relevance, independence, and exclusivity.

Mendelian randomization (MR) is an analytical methodology that employs single-nucleotide polymorphisms (SNPs) that are strongly correlated with exposure variables as instrumental variables (IVs), facilitating inference on the causal relationship between exposures and outcomes. For MR analyses to be valid, they must satisfy three foundational assumptions: (1) a clear association exists between the IVs and the exposure; (2) the IVs are independent of any confounders potentially affecting the exposure and the outcome; and (3) the IVs influence the outcome solely through the exposure, not via any other pathways. This study employed a two-sample MR approach to bidirectionally validate the causal link between inflammatory markers and urolithiasis, including upper and lower urinary tract stones. This design mitigates the influences of confounders and reverse causation biases. Since the data used are publicly available, ethical review and informed consent acquisition for this research were unnecessary. The research framework is illustrated in Figure 1 (11).

### 2.2 Summary statistics source

Initially, we procured a comprehensive meta-analysis dataset on 41 cytokines from The University of Bristol’s official website.^1^ This facilitated the identification of SNPs associated with inflammatory markers. The meta-analysis encompassed 8,293 participants of European ancestry. Notably, the statistical summary data from this meta-analysis did not account for body mass index (BMI) as a model covariate (12). We selected inflammatory markers that did not incorporate BMI as an IV since evidence suggests a potential link between elevated BMI and a heightened risk of urolithiasis (13). Furthermore, we leveraged GWAS data from the latest R9 release in the FinnGen database, extracting information related to CALCUKIDUR (renal and ureteral stones) and CALCULOWER (lower urinary tract stones). These ailments are denoted by ICD-10 codes N20 and N21, respectively. After exclusion of participants with ambiguous gender, high genotyping failure rates (>5%), excessive heterozygosity (±4 SD), and non-Finnish ancestry, the CALCUKIDUR and CALCULOWER samples comprised 9,713 and 1,398 cases, respectively. There were 366,693 controls for both diseases. Additionally, association tests were conducted, adjusting for age, gender, principal genetic components, and genotyping batches. As mentioned earlier, all individuals who withdrew consent were excluded from the data.

### 2.3 Selection of genetic instrumental variables

To pinpoint potential IVs, we primarily selected SNPs intricately associated with inflammatory markers exhibiting genome-wide significance (*p* < 5 × 10^−8^) (14). We set SNP selection thresholds at *R*^2^ < 0.001 and kb = 10,000 to obviate linkage disequilibrium. Only 10 inflammatory cytokines possessed two or more independent SNPs at the *p* < 5 × 10^−8^ level. Consequently, we adjusted the threshold to *p* < 5 × 10^−6^ to identify appropriate IVs. According to this criterion, 41 inflammatory markers were identified. Given the reduced significance threshold, IVs with an *F*-statistic less than ten were deemed weak and excluded (15) (The method for *F*-statistic computations is shown in Supplementary Table S1). To rigorously uphold MR principles, we verified target SNPs via http://www.phenoscanner.medschl.cam.ac.uk/, eliminating outcome-related SNPs. Ultimately, we aligned effect alleles for exposure and outcomes, utilizing MR pleiotropy residual sum and outlier (MR-PRESSO) to detect and exclude IVs with potential horizontal pleiotropy. Detailed SNP listings are available in Supplementary Tables S1–S42.

### 2.4 Mendelian randomization analysis

Our study predominantly employed the inverse variance weighted (IVW) method to gauge the causal relationship between exposures and outcomes. This approach requires that the selected SNPs stringently adhere to the three tenets of MR research, ensuring precise causal estimates (16). When all selected SNPs function as valid IVs, this method offers the most accurate estimate, showcasing relatively high sensitivity to pleiotropy (17). We also implemented the weighted median (WM) and MR-Egger regression methods as a sensitivity measure. WM utilizes median MR estimates for causal estimation, demonstrating greater type I error tolerance than MR-Egger regression, and provides reliable causal estimates even when 50% of the IVs are invalid. The intercept from MR-Egger regression ascertains the presence of horizontal pleiotropy (with intercept *p* < 0.05 considered significant (18)), suggesting that the IVs’ effect on prognosis is independent of the exposure, counter to the definition of IVs. Subsequently, sensitivity analyses were performed. The robustness of the results is detailed in Table 1. We utilized the Cochran’s *Q* test to appraise heterogeneity among SNPs, which is also described in Table 1. A random effects model was adopted in cases of heterogeneity (*p* < 0.05), or SNPs with the smallest *p*-values were discarded to evaluate MR effects. The final “leave-one-out” analysis served to verify the robustness of the results, as detailed in Supplementary Figures S1–S11. All MR analyses were executed using the “TwoSampleMR” package and “MR-PRESSO” package in R version 4.3.1.

**Table 1:** Heterogeneity and multiple effects tests for IVW and MR-Egger analyses.

Exposure	Outcome	Methods	Cochran’s *Q*	*Q*-pval	*p*-value (Pleiotropy test)
IL-2	CALCUKIDUR	MR-Egger	3.588	0.610	0.869
		Inverse variance weighted	3.618	0.728	
MIP-1β	CALCULOWER	MR-Egger	9.917	0.935	0.747
		Inverse variance weighted	10.024	0.952	
SCGF-β	CALCULOWER	MR-Egger	7.373	0.965	0.849
		Inverse variance weighted	7.411	0.978	
CALCUKIDUR	CTACK	MR-Egger	13.586	0.138	0.836
		Inverse variance weighted	13.654	0.189	
CALCUKIDUR	IL-2	MR-Egger	9.608	0.383	0.521
		Inverse variance weighted	10.085	0.433	
CALCULOWER	GRO-α	MR-Egger	7.660	0.363	0.820
		Inverse variance weighted	7.722	0.461	
CALCULOWER	IL-5	MR-Egger	4.768	0.688	0.664
		Inverse variance weighted	4.974	0.760	
CALCULOWER	IL-7	MR-Egger	7.976	0.335	0.842
		Inverse variance weighted	8.025	0.431	
CALCULOWER	IL-8	MR-Egger	7.917	0.340	0.717
		Inverse variance weighted	8.078	0.426	
CALCULOWER	MIG	MR-Egger	9.495	0.219	0.453
		Inverse variance weighted	10.351	0.241	
CALCULOWER	MIP-1α	MR-Egger	6.261	0.510	0.454
		Inverse variance weighted	6.889	0.549	

## 3 Results

### 3.1 Inflammatory factors and renal stone disease

**Figure 2:**
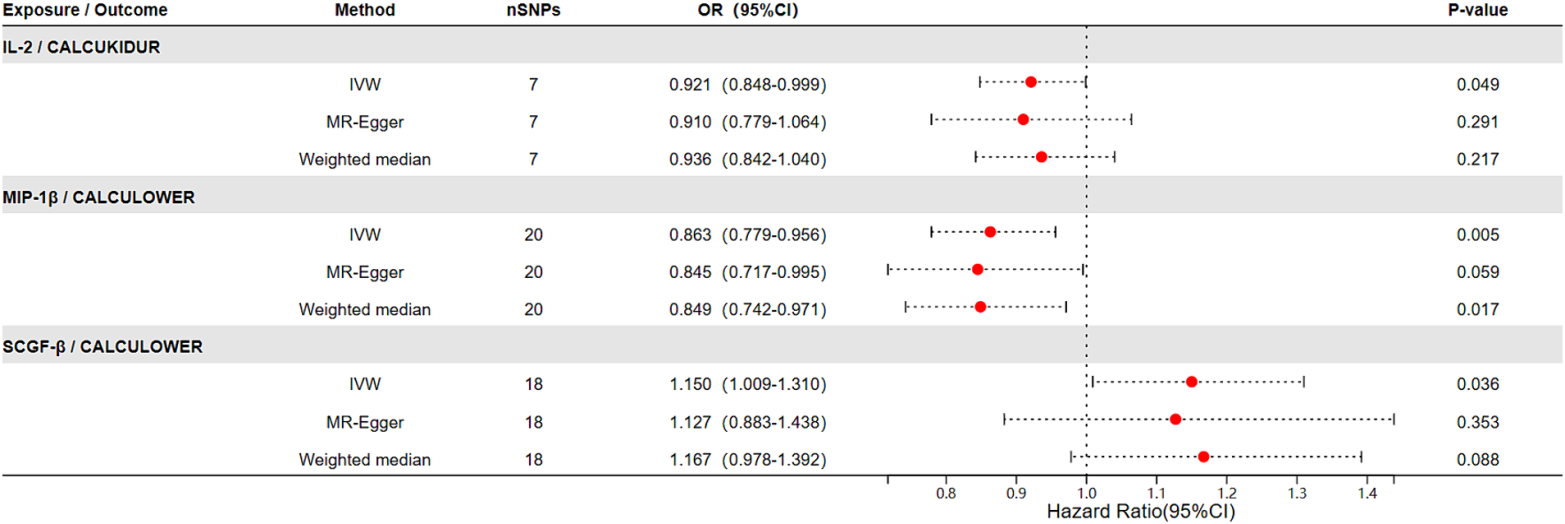
The relationship between genetically predicted inflammatory factors and the risk of urolithiasis (upper urinary tract stones and lower urinary tract stones) (IVW, MR-Egger, Weighted median).

The results of the Mendelian randomization (MR) study of genetically predicted inflammatory markers are associated with renal stone disease, encompassing IVW, MR-Egger, and weighted median methods, are illustrated in Figure 2. Our analyses indicate that elevated levels of IL-2 significantly reduce the risk of renal stone disease (OR = 0.921, 95% CI = 0.848–0.999). The MR-Egger intercept test did not detect significant horizontal pleiotropy (*p*-value > 0.05). Cochrane’s *Q* test also revealed no significant heterogeneity (*p*-value > 0.05). Additionally, heterogeneity tests for IVW and MR-Egger did not exhibit notable heterogeneity (*p*-value > 0.05). Sensitivity analyses employing the leave-one-out method did not discern any significant impact, as shown in Supplementary Figure S1.

### 3.2 Inflammatory factors and lower urinary tract stones

In the outcomes studied of lower urinary tract stones, we noticed high levels of MIP-1β were associated with a decreased risk of lower urinary tract stones (OR = 0.863, 95% CI = 0.779–0.956). Moreover, elevated levels of SCGF-β correlated with an increased risk of lower urinary tract stones (OR = 1.150, 95% CI = 1.009–1.310), with the results displaying no heterogeneity or horizontal pleiotropy (*p* > 0.05) (Figure 2). Cochran’s *Q* test did not found heterogeneity. Similarly, MR-PRESSO did not find any abnormal SNPs. Meanwhile, our results’ reliability was verified by both the MR-Egger intercept technique and the leave-one-out method (Supplementary Figures S2, S3).

### 3.3 Reverse causal association of inflammatory factors and renal stone disease

In reverse validation, we observed correlations between genetically predicted urolithiasis and cytokine levels, as detailed in Table 2. Using 11 SNPs highly associated with renal stone disease as IVs (*p* < 5 × 10^−8^, discounting linkage disequilibrium with *R*^2^ < 0.001, kb = 10,000), our IVW methodology demonstrated that genetically predicted renal stone disease was correlated with CTACK (*β* = 0.208, 95% CI = 0.058–0.357) and IL-2 (*β* = 0.145, 95% CI = 0.013–0.276) levels. The results did not demonstrate polyvalence and heterogeneity (Tables 1, 2 and Supplementary Figures S4, S5).

**Table 2:** The relationship between genetic prediction of upper and lower urinary tract stone and inflammatory factor risks (IVW, MR-Egger, WM).

Exposure	Outcome	Methods	Number of SNPs	OR (95% CI)	*p*-value
		IVW	11	0.208 (0.058–0.357)	0.006
CCALCUKIDUR	CTACK	MR-Egger	11	0.160 (−0.300–0.621)	0.512
		Weighted median	11	0.130 (−0.055–0.314)	0.168
		IVW	11	0.145 (0.013–0.276)	0.031
CCALCUKIDUR	IL-2	MR-Egger	11	0.272 (−0.125–0.669)	0.212
		Weighted median	11	0.162 (−0.026–0.350)	0.092
		IVW	9	0.142 (0.057–0.226)	0.001
CALCULOWER	IL-5	MR-Egger	9	0.178 (0.001–0.354)	0.089
		Weighted median	9	0.155 (0.036–0.275)	0.011
		IVW	9	0.108 (0.024–0.192)	0.012
CALCULOWER	IL-7	MR-Egger	9	0.125 (−0.061–0.311)	0.229
		Weighted median	9	0.019 (0.255–1.147)	0.023
		IVW	9	0.127 (0.044–0.210)	0.003
CALCULOWER	IL-8	MR-Egger	9	0.096 (−0.086–0.278)	0.335
		Weighted median	9	0.102 (−0.016–0.219)	0.090
		IVW	9	0.099 (0.008–0.191)	0.033
CALCULOWER	MIG	MR-Egger	9	0.030 (−0.165–0.225)	0.772
		Weighted median	9	0.117 (0.006–0.228)	0.039
		IVW	9	0.126 (0.044–0.208)	0.003
CALCULOWER	MIP-1α	MR-Egger	9	0.066 (−0.105–0.236)	0.476
		Weighted median	9	0.112 (−0.001–0.226)	0.052
		IVW	9	0.086 (0.004–0.169)	0.040
CALCULOWER	GRO-α	MR-Egger	9	0.067 (−0.112–0.246)	0.485
		Weighted median	9	0.068 (−0.046–0.182)	0.241

### 3.4 Reverse causal association of inflammatory factors and lower urinary tract stones

No conforming SNPs were identified when selecting SNPs that were highly correlated with lower urinary tract stones (employing 5 × 10^−8^ as a selection parameter). Consequently, we adjusted our selection threshold to a more permissive criterion of 5 × 10^−6^ while still accounting for eliminating linkage disequilibrium (*R*^2^ < 0.001, kb = 10,000). We identified 9 SNPs as IVs for lower urinary tract stones. MR analyses using IVW demonstrated that genetically predicted lower urinary tract stones correlated with the levels of several cytokines, such as GRO-α, IL-5, IL-7, IL-8, MIG, and MIP-1α. None of the results exhibited any significant pleiotropy or heterogeneity. The pertinent data are consolidated in Tables 1, 2. The leave-one-out analyses, scatter plots, funnel plots, and forest plots are all included in the Supplementary material.

## 4 Discussion

Urolithiasis has emerged as a significant global public health concern, with prevalence rates estimated between 1 and 15%, that is influenced by factors such as geography, diet, socioeconomic status, and genetics. Its prevalence has been on a consistent rise (2). Renal calculi are the most prevalent. Although surgical interventions have rapidly evolved over the years and increased the cure rate, the recurrence of renal stones remains concerning. Current preventive measures predominantly revolve around medication that alters urinary chemistry to reduce crystal supersaturation. This urges a deeper investigation into the pathophysiology and etiology of stone formation. Both *in vivo* and *in vitro* studies underscore the formation of Randall’s plaques as crucial in the development of idiopathic calcium oxalate stones (19, 20). Interestingly, a retrospective cohort study found a link between statin (anti-inflammatory and cholesterol-lowering drugs) use and a reduced risk of stone formation after adjusting for age, sex, and comorbidities (21).

Moreover, many proteins associated with inflammatory cells and processes have been identified in calcium oxalate stones (22, 23). Proinflammatory cytokines such as TNF, IL-1β, and IL-6 have been observed at notably higher levels in patients with renal stones than in those without renal stones (24). Animal studies have indicated the potential role of CSF-1 signaling in suppressing renal crystal formation by promoting the polarization of macrophages toward the M2 phenotype (8). These observational studies indicate a potential link between inflammation and renal stone formation. However, these observational results warrant further exploration due to the possibility of reverse causation or confounding factors; whether inflammation precedes stone formation or vice versa remains enigmatic. Thus, a comprehensive understanding of the pathogenesis of renal stones is pivotal for more effective prevention of recurrence.

MR provides a unique opportunity to overcome common confounders and biases in traditional observational studies, offering more substantial evidence for determining causative associations (9). Our study used this method to better understand the potential relationships between genetically predisposed inflammatory cytokines and urolithiasis. The findings of MR analyses using 41 different cytokines as IVs reveal intriguing associations. Specifically, an elevation in circulating IL-2 seems to be associated with an increased risk of renal stone disease. Furthermore, our observations linked elevated levels of SCGF-β and reduced levels of MIP-1β to a heightened risk of lower urinary tract stones.

Recent studies indicate that the formation of Randall’s plaques and idiopathic stones is not solely a result of renal tubular urine supersaturation-induced calcification but involves inflammation and immune responses during plaque formation and lithogenesis (25). Observing the pathological similarities between Randall’s plaques and atherosclerosis, it is interesting to note that combined treatment using CD3 and IL-2 has been demonstrated to inhibit the formation of atherosclerotic plaques (26, 27), leading to a pertinent question: Could a similar immunoregulatory strategy also effectively control the progression of Randall’s plaques?

In our MR study, an elevated peripheral level of IL-2 appeared to be associated with a reduced risk of renal stone disease. Unfortunately, there are few observational reports on the relationship between peripheral IL-2 and renal stone disease. IL-2, a critical cytokine, is pivotal in inflammation and immune responses. By modulating Tregs and optimizing the response of effector lymphocytes, IL-2 aids in maintaining immunological balance and forestalling autoimmune reactions. Considering this perspective, crystals could potentially harm renal tubular epithelial cells under conditions of urine supersaturation, triggering an elevation in IL-2 levels; this then stimulates the activation of Tregs and polarization of M2 macrophages, attenuating the initial inflammatory response or renal injury and subsequently reducing stone formation risks. However, in scenarios where inflammation persists accompanied by urine supersaturation, immune responses could become dysregulated, instigating the formation of Randall’s plaques and eventually leading to stone formation. In this context, the potential of IL-2 as a biomarker merits further investigation.

Our study also indicates that an elevated peripheral level of MIP-1β reduces the risk of lower urinary tract stones. Although the etiology of lower urinary tract stones is diverse (28), a proteomic-based study has identified a potential role of inflammatory reactions in forming bladder uric acid stones (29). MIP-1β, also known as CCL4, is a chemokine that plays a role in immune system inflammatory responses (30). Bladder inflammation could alter the pH of urine, resulting in an increased presence of inflammatory cells and debris (31). Moreover, inflammation might impact the bladder’s ability to contract and void completely, creating conditions conducive to mineral deposition and stone formation. The exact role of MIP-1β in lower urinary tract stones, with its chemotactic properties that transition the inflammatory process from pro-inflammatory to anti-inflammatory, remains to be thoroughly explored.

Another cytokine of interest, SCGF-β, is a crucial regulator of early hematopoiesis (32). Our MR findings link elevated levels of SCGF-β with an increased risk of lower urinary tract stones. No studies have focused on the role of SCGF-β in lower urinary tract stones. The precise mechanism and function of SCGF-β in inflammation remain largely unknown and could vary depending on the environment and interactions with other cytokines and signaling molecules.

Through bidirectional MR analyses, we found potential links between urolithiasis and alterations in circulating levels of specific cytokines. Specifically, our data indicate that renal stone disease correlates significantly with elevated levels of IL-2 and CTACK, and these findings displayed neither significant heterogeneity nor horizontal pleiotropy. Stones in the kidneys or ureters might cause upper urinary tract obstructions, leading to injuries in the renal tubules and interstitium, initiating inflammatory cell infiltration and interstitial fibrosis (33). This physiological response might activate T cells and other immune cells, potentially explaining the rise in IL-2 levels since IL-2 is a product of activated T cells,. Concurrently, CTACK might act as a chemokine, recruiting anti-inflammatory immune cells such as Tregs and M2 macrophages, which are pivotal in mitigating inflammation and promoting tissue repair.

In the context of lower urinary tract stones, our bidirectional MR analyses unveiled elevations in various cytokines, including GRO-α, IL-5, IL-7, IL-8, MIG, and MIP-1α. These cytokines play core roles in recruiting, activating, and modulating neutrophils, T cells, and other immune cells. Given the association of lower urinary tract stone disease with elevated levels of multiple cytokines, we hypothesize that these cytokines might play a protective role during stone-induced inflammatory responses. These discoveries bolster the significance of inflammatory cytokine-regulated immune reactions in the pathogenesis of urolithiasis. Further studies are warranted to elucidate the molecular pathways of these alterations in inflammatory cytokines and their roles in urolithiasis onset, progression, treatment strategies, and recurrence prevention.

Our study’s strength lies in employing the MR method to investigate the relationship between systemic inflammatory regulators and urolithiasis, leveraging the data from extensive GWAS meta-analyses. We ensured the robustness of our instrumental variables through multiple heterogeneity and pleiotropy tests, thus minimizing the effects of reverse causality and potential confounders. However, some limitations persist.

First, for the GWAS data of inflammatory cytokines, we used a *p*-value threshold of <5 × 10^−6^ for significance, as a stricter criterion of <5 × 10^−8^ resulted in very few factors meeting the threshold. Second, all the GWAS data are derived from European populations; thus, the applicability of our findings in other demographics remains uncertain. Furthermore, we did not stratify our analysis according to different stone compositions due to the absence of detailed data on stone compositions. Finally, intricate interactions exist between urolithiasis and the inflammatory response. In addition to the disease itself influencing the production of inflammatory cytokines, other unconsidered confounding factors, such as networks of inflammatory factors, might be in play. Therefore, this field necessitates more profound and comprehensive research.

## 5 Conclusion

In conclusion, our research supports the evidence that genetically determined levels of IL-2 are associated with a reduced risk of renal stone formation and that levels of SCGF-β and MIP-1β are correlated with the risk of lower urinary tract stones. This suggests that modulation and intervention of certain inflammatory factors could be effective strategies for the prevention of urolithiasis in the future.

## Data availability statement

The datasets presented in this study can be found in online repositories. The names of the repository/repositories and accession number(s) can be found in the article/Supplementary material.

## Ethics statement

For this study, ethical approval was not required as per institutional and national guidelines. We used publicly available, de-identified data from genome-wide association studies (GWAS) and the FinnGen Consortium R9 dataset. These datasets were analyzed in accordance with the relevant guidelines and regulations for secondary data analysis. No new data were generated, and no human participants were directly involved in the study.

## Author contributions

KH: Conceptualization, Data curation, Methodology, Writing – original draft. ZP: Conceptualization, Data curation, Methodology, Writing – original draft. CZ: Conceptualization, Methodology, Visualization, Writing – original draft. WL: Conceptualization, Writing – original draft. GD: Formal analysis, Software, Writing – original draft. XC: Data curation, Visualization, Writing – original draft. YL: Data curation, Visualization, Writing – original draft. ZJ: Methodology, Visualization, Writing – original draft. QW: Conceptualization, Funding acquisition, Supervision, Writing – review & editing. KJ: Conceptualization, Funding acquisition, Supervision, Writing – review & editing.

## Footnotes

^1^ https://data.bris.ac.uk/data/dataset/3g3i5smgghp0s2uvm1doflkx9x
