# Supplementary material for: Inflammatory factors and the risk of urolithiasis: a bidirectional Mendelian randomization study
Source: Front Med (Lausanne). 2024 Jul 3;11:1432275. doi: 10.3389/fmed.2024.1432275 (PMC11251917; doi:10.3389/fmed.2024.1432275)
Supplement: Supplementary file 1 [file Data_Sheet_1.docx]

**Supplementary Figure 1 Leave-one-out Analysis, Scatter Plot, Funnel Plot, and Forest Plot of** **IL-2 on Kidney Stone**

**
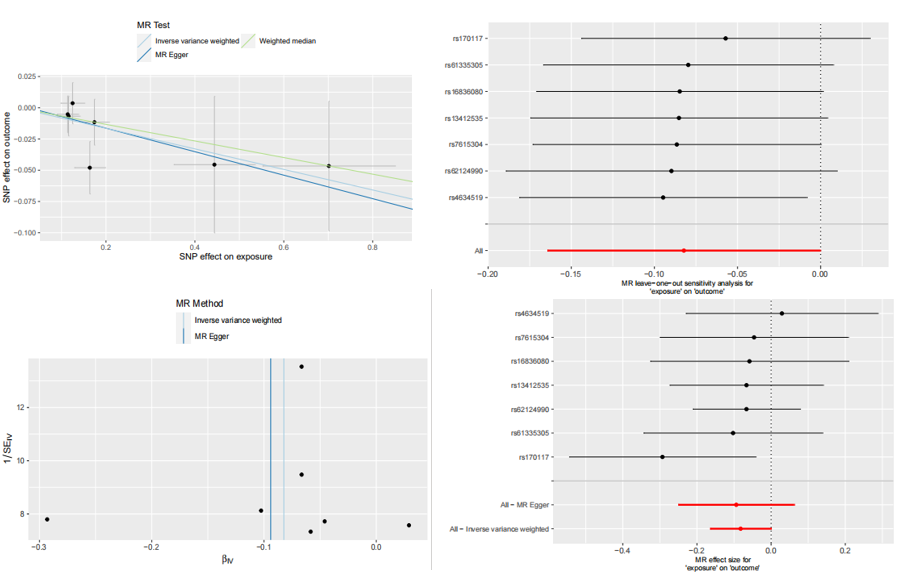
**

**Supplementary Figure 2** **Leave-one-out Analysis, Scatter Plot, Funnel Plot, and Forest Plot of MIP-1β o****n calculus of lower urinary tract**

**
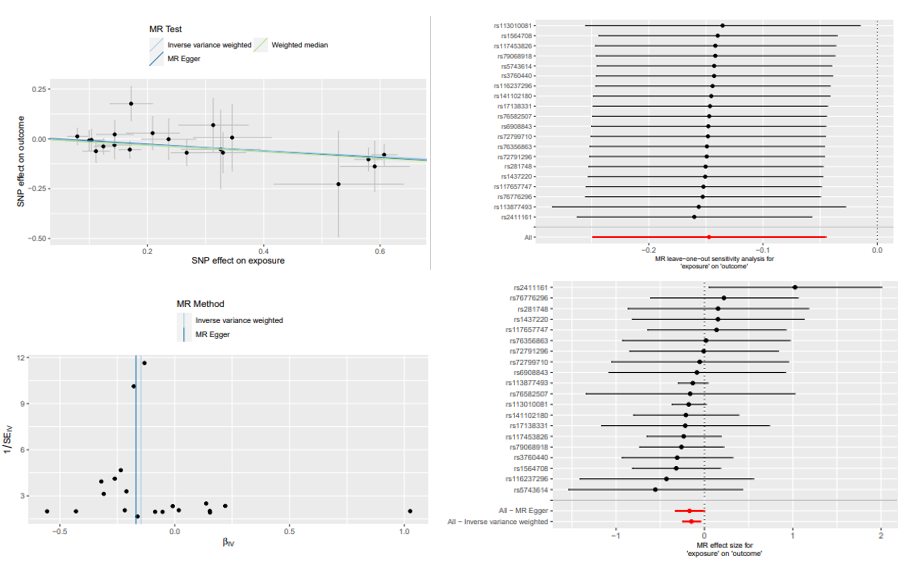
**

**Supplementary Figure 3 Leave-one-out Analysis, Scatter Plot, Funnel Plot, and Forest Plot of SCGF-β on calculus of lower urinary tract**

**
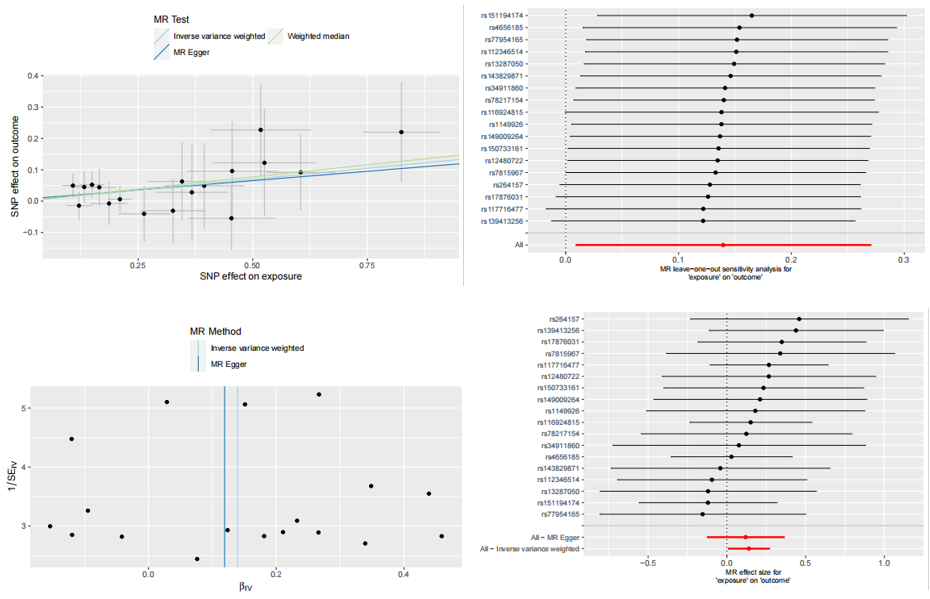
**

**Supplementary Figure 4 Leave-one-out Analysis, Scatter Plot, Funnel Plot, and Forest Plot of Kidney Stones on CTACK**

**
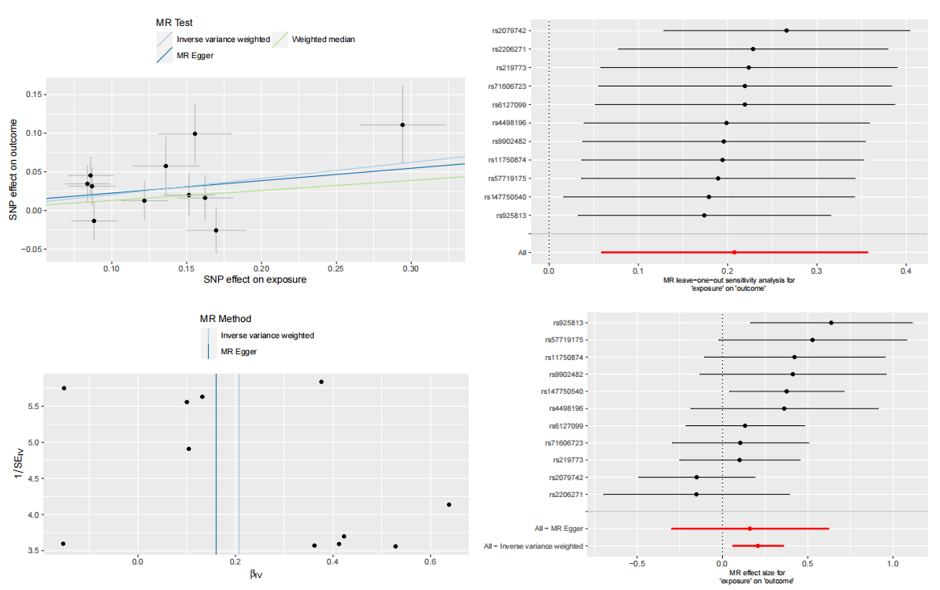
**

**Supplementary Figure 5 Leave-one-out Analysis, Scatter Plot, Funnel Plot, and Forest Plot of Kidney Stones on IL-2**

**
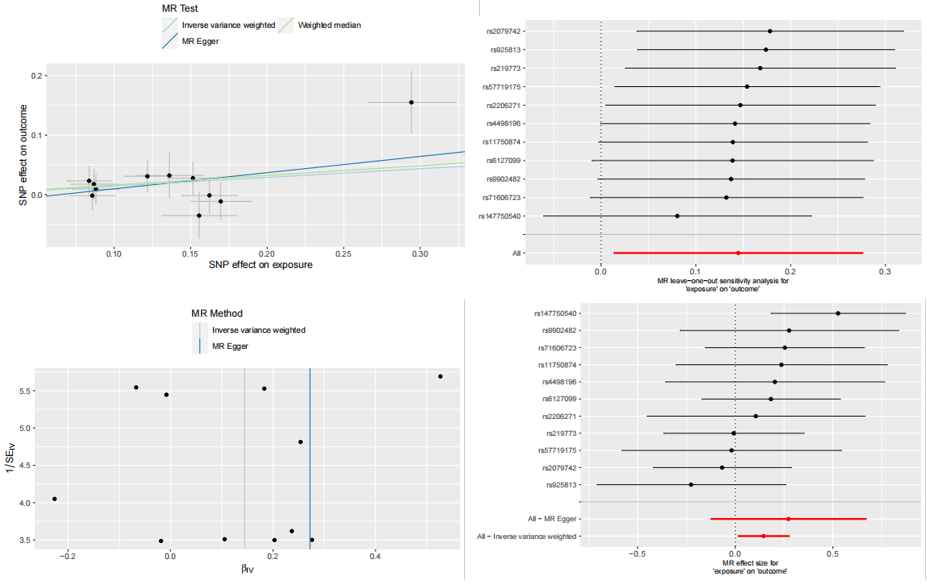
**

**Supplementary Figure 6 Leave-one-out Analysis, Scatter Plot, Funnel Plot, and Forest Plot of calculus of lower urinary tract on GRO-α**

**
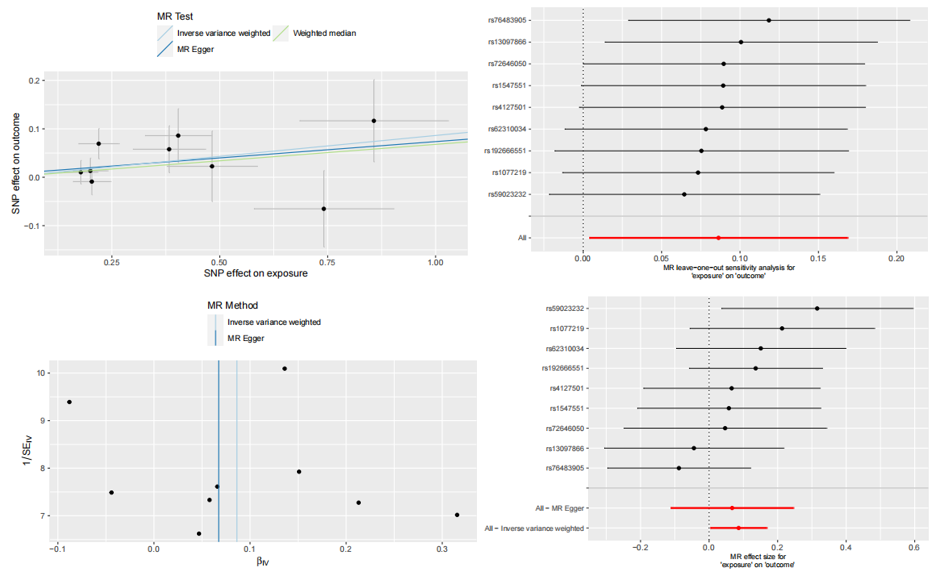
**

**Supplementary Fi****gure 7 Leave-one-out Analysis, Scatter Plot, Funnel Plot, and Forest Plot of calculus of lower urinary tract on IL-5**

**
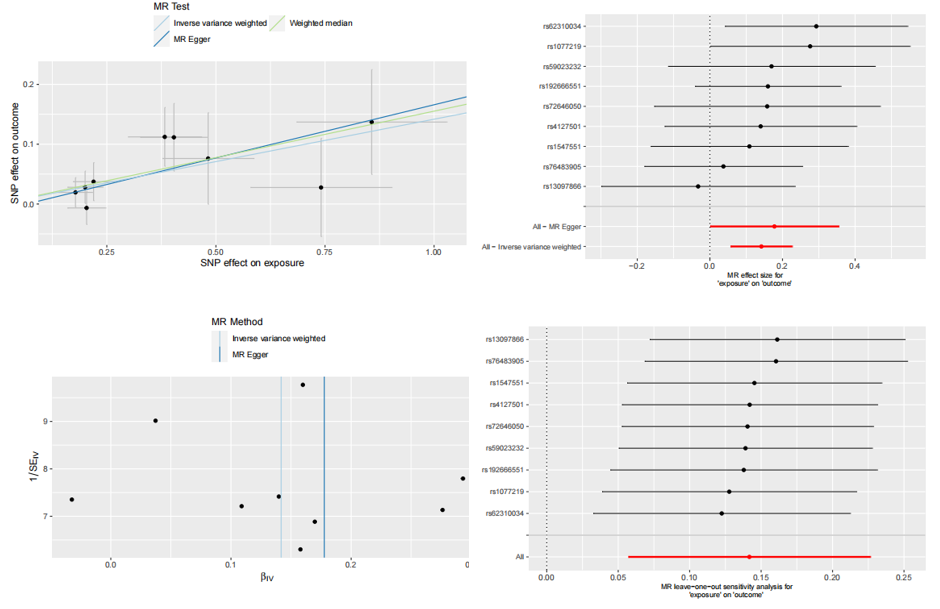
**

**Supplementary Figure 8 Leave-one-out Analysis, Scatter Plot, Funnel Plot, and Forest Plot of calculus of lower urinary tract on IL-7**

**
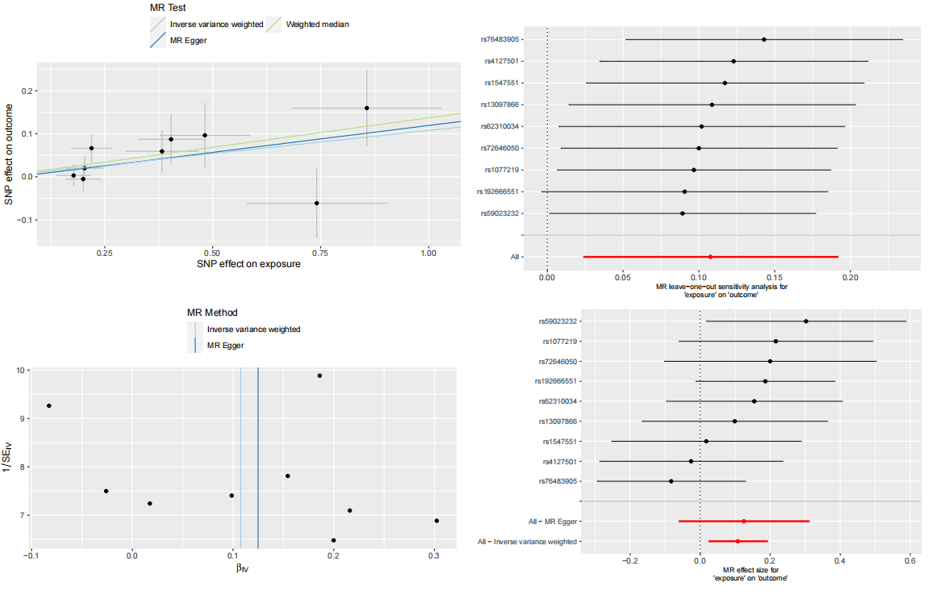
**

**Supplementary Figure 9 Leave-one-out Analysis, Scatter Plot, Funnel Plot, and Forest Plot of calculus of lower urinary tract on IL-8**

**
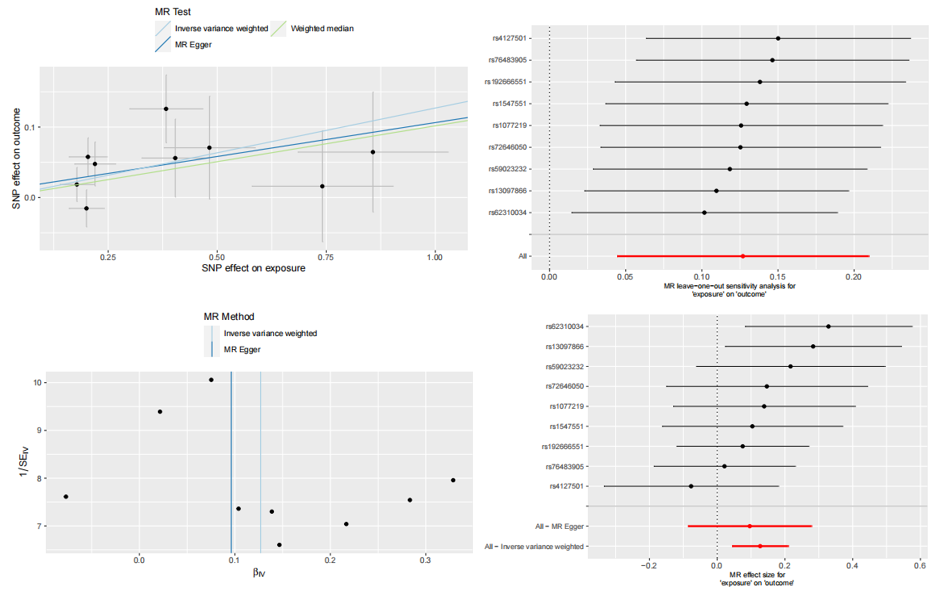
**

**Supplementary Figure 10 Leave-one-out Analysis, Scatter Plot, Funnel Plot, and Forest Plot of calculus of lower urinary tract on MIG**

**
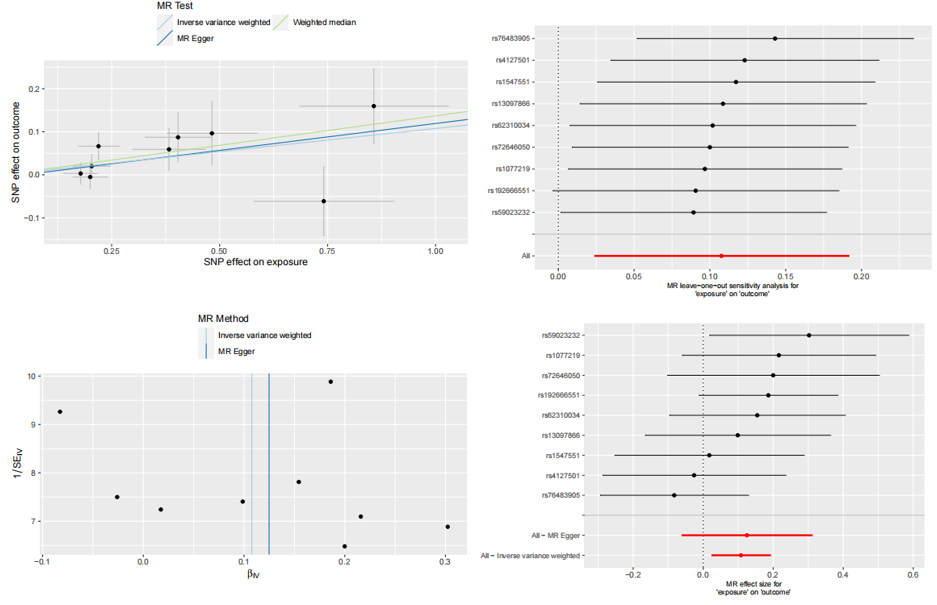
**

**Supplementary Figure 11 Leave-one-out Analysis, Scatter Plot, Funnel Plot, and Forest Plot of calculus of lower urinary tract on MIP-1α**

**
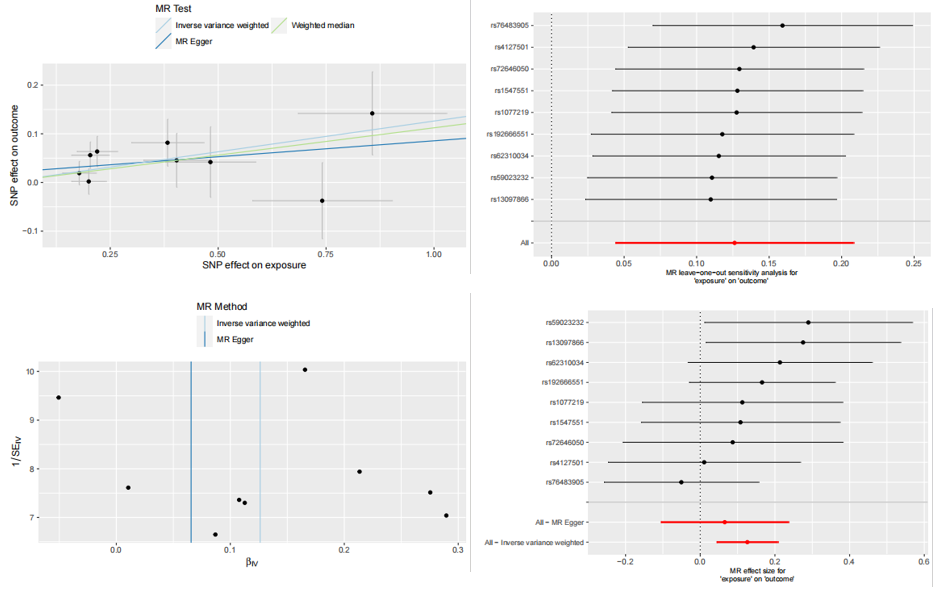
**

**Supplementary Figure 12 The association between genetically determined inflammatory cytokines and the risk of Kidney Stone (except IL-2)**

**
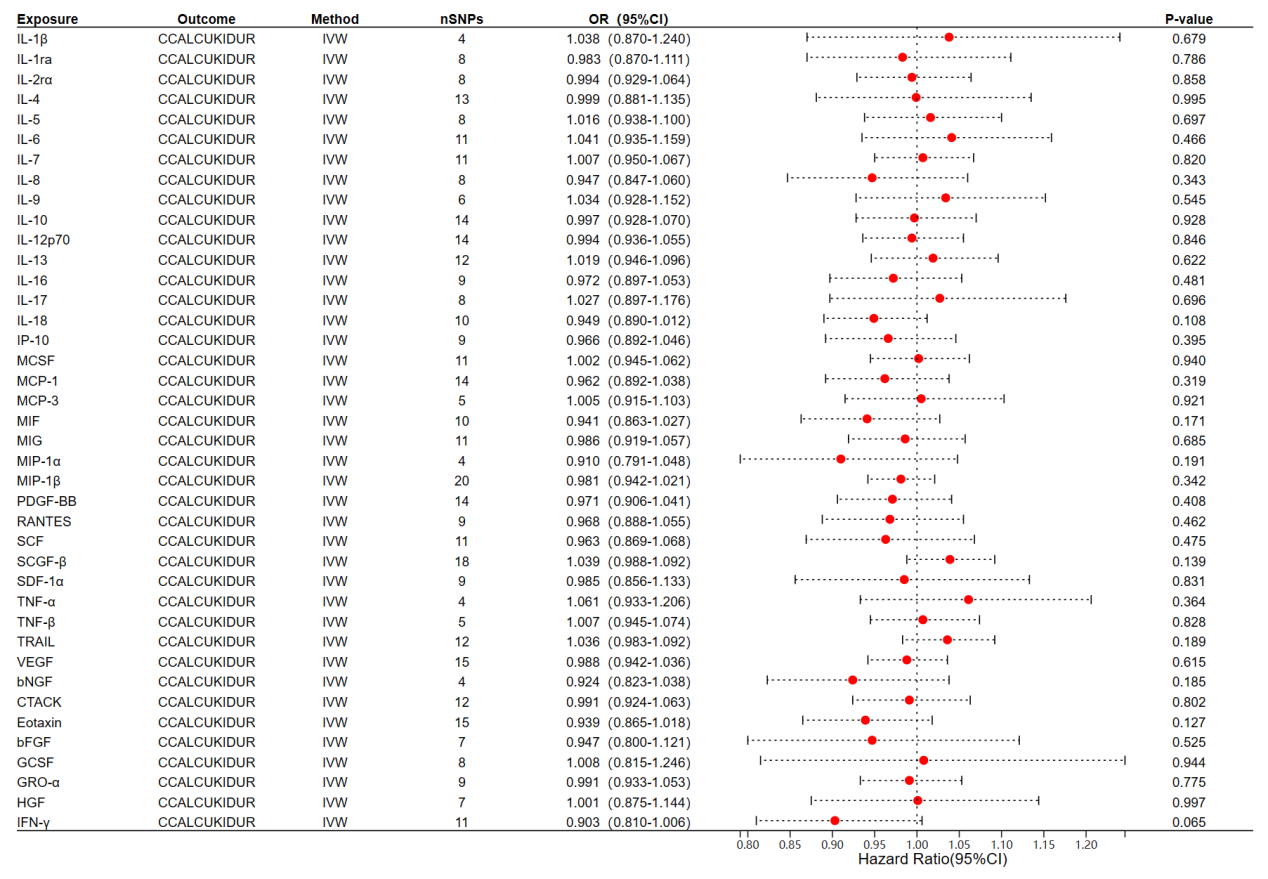
**

CALCUKIDUR：Calculus of kidney and ureter

**Supplementary Figure 13 The association between genetically determined inflammatory cytokines and the risk of calculus of lower urinary tract(except MIP-1β and SCGF-β)**

**
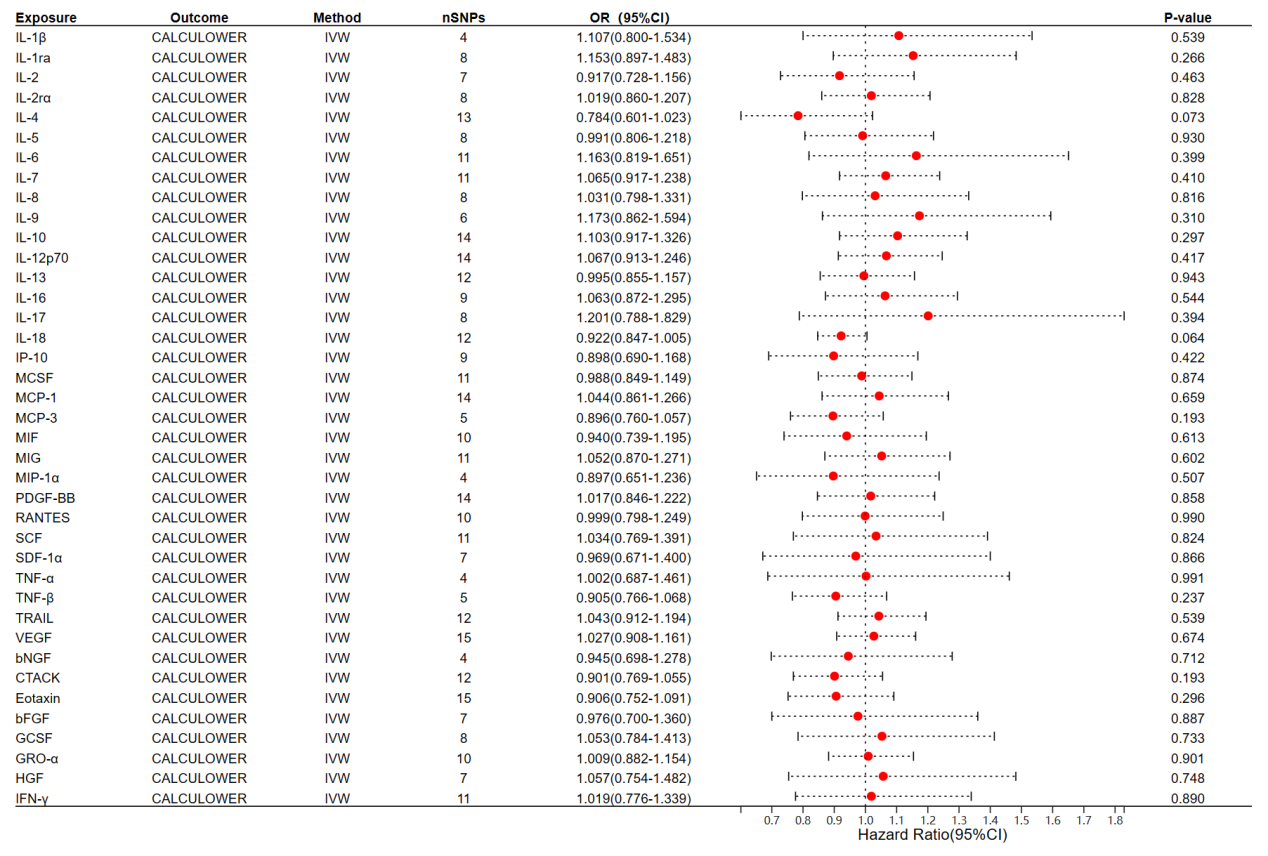
**

CALCULOWER：Calculus of lower urinary tract

**Supplementary Table S1** The method for F-statistic computations

| F =$\frac{R^{2} / k}{(1 - R^{2}) / (n - k - 1)}$  In Mendelian randomisation the first stage $R^{2}$ is the proportion of risk factor variability explained by genotype. K is the number of instrumental variables, and n is the sample size. IVs with an F-statistic less than ten were deemed weak and excluded. |
| --- |

**Supplementary Table 1** Characteristics of the single nucleotide polymorphisms (SNP) used as instrumental variables for beta nerve growth factor (β-NGF)

| **SNP** | **Effect Allele** | **Other Allele** | **β** | **SE** | **P-Value** | **F** |
| --- | --- | --- | --- | --- | --- | --- |
| rs28637706 | T | G | -0.1554 | 0.0261 | 2.717E-09 | 35.431 |
| rs71641308 | T | C | 0.1969 | 0.0429 | 4.424E-06 | 21.054 |
| rs73472576 | T | C | -0.1146 | 0.0251 | 4.813E-06 | 20.834 |
| rs7970581 | T | G | 0.1358 | 0.028 | 1.223E-06 | 23.509 |

**Supplementary Table 2** Characteristics of the single nucleotide polymorphisms (SNP) used as instrumental variables for cutaneous T cell-attracting chemokine (CTACK)

| **SNP** | **Effect Allele** | **Other Allele** | **β** | **SE** | **P-Value** | **F** |
| --- | --- | --- | --- | --- | --- | --- |
| rs116303454 | A | G | 0.3754 | 0.081 | 3.579E-06 | 21.468 |
| rs118084576 | A | G | 0.5675 | 0.1226 | 3.66E-06 | 21.415 |
| rs135564 | A | G | -0.1672 | 0.0267 | 3.593E-10 | 39.193 |
| rs141331414 | A | G | 0.1977 | 0.0415 | 1.886E-06 | 22.682 |
| rs2070074 | A | G | 0.4401 | 0.0372 | 2.596E-32 | 139.888 |
| rs55764737 | T | C | 0.5424 | 0.0967 | 2.012E-08 | 31.444 |
| rs57338032 | A | G | 0.1443 | 0.0316 | 4.831E-06 | 20.841 |
| rs60247384 | T | C | 0.1128 | 0.0245 | 4.302E-06 | 21.186 |
| rs62578137 | T | C | -0.1311 | 0.0286 | 4.658E-06 | 21.001 |
| rs72729450 | T | C | -0.5123 | 0.1094 | 2.814E-06 | 21.917 |
| rs7333764 | T | C | 0.2811 | 0.0591 | 2.002E-06 | 22.610 |
| rs76395525 | A | G | 0.5193 | 0.1081 | 1.553E-06 | 23.065 |

**Supplementary Table 3** Characteristics of the single nucleotide polymorphisms (SNP) used as instrumental variables for CCL11 (Eotaxin)

| **SNP** | **Effect Allele** | **Other Allele** | **β** | **SE** | **P-Value** | **F** |
| --- | --- | --- | --- | --- | --- | --- |
| rs11087905 | A | C | 0.0954 | 0.0188 | 4.07E-07 | 25.744 |
| rs112347425 | T | C | 0.1595 | 0.0276 | 7.771E-09 | 33.389 |
| rs12075 | A | G | 0.1692 | 0.0155 | 1.211E-27 | 119.132 |
| rs147287945 | A | G | -0.1512 | 0.0313 | 1.356E-06 | 23.330 |
| rs187131 | C | G | 0.1264 | 0.0253 | 5.742E-07 | 24.954 |
| rs2024050 | A | G | 0.164 | 0.0302 | 5.467E-08 | 29.483 |
| rs2027855 | T | C | 0.0743 | 0.0162 | 4.272E-06 | 21.030 |
| rs2211994 | T | C | 0.0876 | 0.0177 | 6.981E-07 | 24.488 |
| rs2228467 | T | C | -0.4154 | 0.0291 | 3.473E-46 | 203.723 |
| rs5754733 | A | C | -0.105 | 0.0213 | 8.196E-07 | 24.295 |
| rs59808887 | T | C | -0.1698 | 0.0356 | 1.886E-06 | 22.744 |
| rs745331 | A | G | -0.0821 | 0.0176 | 3.036E-06 | 21.755 |
| rs75426604 | A | C | -0.1371 | 0.0291 | 2.397E-06 | 22.191 |
| rs79722574 | T | C | -0.1092 | 0.0227 | 1.497E-06 | 23.136 |
| rs9317045 | A | C | 0.1172 | 0.0236 | 6.954E-07 | 24.656 |

**Supplementary Table 4** Characteristics of the single nucleotide polymorphisms (SNP) used as instrumental variables for basic fibroblast growth factor (bFGF)

| **SNP** | **Effect Allele** | **Other Allele** | **β** | **SE** | **P-Value** | **F** |
| --- | --- | --- | --- | --- | --- | --- |
| rs116745220 | A | G | -0.6176 | 0.1324 | 3.085E-06 | 21.753 |
| rs13412535 | A | G | -0.1129 | 0.0224 | 4.763E-07 | 25.397 |
| rs145577605 | A | G | 0.2043 | 0.0427 | 1.672E-06 | 22.886 |
| rs61990749 | C | G | 0.1124 | 0.0228 | 8.225E-07 | 24.297 |
| rs75168112 | T | C | -0.1024 | 0.0214 | 1.637E-06 | 22.890 |
| rs78873483 | A | G | 0.1286 | 0.0282 | 4.98E-06 | 20.791 |
| rs9903590 | T | C | 0.1281 | 0.0267 | 1.622E-06 | 23.012 |

**Supplementary Table 5** Characteristics of the single nucleotide polymorphisms (SNP) used as instrumental variables for granulocyte colony-stimulating factor (G-CSF)

| **SNP** | **Effect Allele** | **Other Allele** | **β** | **SE** | **P-Value** | **F** |
| --- | --- | --- | --- | --- | --- | --- |
| rs115256310 | A | G | -0.6788 | 0.1359 | 5.85E-07 | 24.942 |
| rs117261691 | T | C | 0.1318 | 0.0288 | 4.669E-06 | 20.938 |
| rs11903143 | A | G | 0.0889 | 0.0175 | 3.784E-07 | 25.800 |
| rs145756094 | C | G | -0.7323 | 0.1479 | 7.399E-07 | 24.509 |
| rs2671444 | A | G | -0.0776 | 0.0166 | 2.861E-06 | 21.847 |
| rs586313 | T | C | -0.0883 | 0.0187 | 2.355E-06 | 22.291 |
| rs74148555 | T | C | -0.3771 | 0.0753 | 5.591E-07 | 25.073 |
| rs77318030 | T | C | -0.2031 | 0.0427 | 2.017E-06 | 22.618 |

**Supplementary Table 6** Characteristics of the single nucleotide polymorphisms (SNP) used as instrumental variables for growth regulated oncogene alpha (GRO-α)

| **SNP** | **Effect Allele** | **Other Allele** | **β** | **SE** | **P-Value** | **F** |
| --- | --- | --- | --- | --- | --- | --- |
| rs1113500 | T | G | 0.1162 | 0.0243 | 1.721E-06 | 22.854 |
| rs114991247 | T | C | -0.2202 | 0.0463 | 1.971E-06 | 22.606 |
| rs12075 | A | G | 0.3724 | 0.0236 | 3.459E-56 | 248.857 |
| rs140734053 | A | G | 0.7333 | 0.1545 | 2.069E-06 | 22.514 |
| rs185768063 | A | G | 0.4038 | 0.076 | 1.055E-07 | 28.214 |
| rs188345231 | T | C | 0.6177 | 0.1322 | 2.968E-06 | 21.820 |
| rs508977 | T | G | -0.3838 | 0.0279 | 4.566E-43 | 189.128 |
| rs62024303 | A | G | -0.3013 | 0.066 | 4.908E-06 | 20.829 |
| rs76390238 | C | G | 0.6223 | 0.1352 | 4.141E-06 | 21.174 |
| rs78653452 | T | G | -0.7395 | 0.1559 | 2.093E-06 | 22.487 |

（Rs185768063 was found to have horizontal pleiotropy by MR-PRESSO of GRO-α on Kidney Stone, so it was removed.）

**Supplementary Table 7** Characteristics of the single nucleotide polymorphisms (SNP) used as instrumental variables for hepatocyte growth factor (HGF)

| **SNP** | **Effect Allele** | **Other Allele** | **β** | **SE** | **P-Value** | **F** |
| --- | --- | --- | --- | --- | --- | --- |
| rs11060254 | A | G | -0.0765 | 0.0166 | 3.974E-06 | 21.233 |
| rs13412535 | A | G | -0.1043 | 0.0213 | 9.671E-07 | 23.972 |
| rs1617833 | C | G | -0.0749 | 0.016 | 2.925E-06 | 21.909 |
| rs2003620 | T | C | 0.2277 | 0.0487 | 2.978E-06 | 21.856 |
| rs3748034 | T | G | 0.1529 | 0.0233 | 5.21E-11 | 43.053 |
| rs4245058 | T | C | -0.1552 | 0.0331 | 2.683E-06 | 21.980 |
| rs5745687 | T | C | -0.3008 | 0.0404 | 9.922E-14 | 55.423 |

**Supplementary Table 8** Characteristics of the single nucleotide polymorphisms (SNP) used as instrumental variables for interferon gamma (IFN-γ)

| **SNP** | **Effect Allele** | **Other Allele** | **β** | **SE** | **P-Value** | **F** |
| --- | --- | --- | --- | --- | --- | --- |
| rs10481651 | A | G | -0.0793 | 0.0168 | 2.183E-06 | 22.275 |
| rs10761731 | A | T | -0.0813 | 0.0167 | 1.068E-06 | 23.694 |
| rs113600793 | A | C | 0.1871 | 0.0371 | 4.426E-07 | 25.427 |
| rs115729819 | A | G | 0.2511 | 0.0514 | 1.045E-06 | 23.859 |
| rs11843756 | T | G | 0.1812 | 0.0391 | 3.622E-06 | 21.471 |
| rs12420286 | T | C | 0.2357 | 0.05 | 2.452E-06 | 22.216 |
| rs2073438 | A | G | 0.092 | 0.0188 | 9.551E-07 | 23.941 |
| rs2188420 | C | G | 0.1005 | 0.0201 | 5.898E-07 | 24.994 |
| rs60059008 | A | G | 0.0852 | 0.0176 | 1.301E-06 | 23.428 |
| rs74148555 | T | C | -0.3771 | 0.077 | 9.858E-07 | 23.978 |
| rs78296352 | T | G | 0.3419 | 0.065 | 1.42E-07 | 27.660 |

**Supplementary Table 9** Characteristics of the single nucleotide polymorphisms (SNP) used as instrumental variables for interleukin-1 beta (IL-1β)

| **SNP** | **Effect Allele** | **Other Allele** | **β** | **SE** | **P-Value** | **F** |
| --- | --- | --- | --- | --- | --- | --- |
| rs115242021 | A | C | 0.2795 | 0.0553 | 4.252E-07 | 25.530 |
| rs143319329 | T | C | 0.4357 | 0.093 | 2.835E-06 | 21.936 |
| rs61335305 | A | C | 0.4333 | 0.0928 | 3.015E-06 | 21.788 |
| rs62015704 | A | G | 0.1786 | 0.0372 | 1.624E-06 | 23.037 |

**Supplementary Table 10** Characteristics of the single nucleotide polymorphisms (SNP) used as instrumental variables for interleukin-1 receptor antagonist (IL-1ra)

| **SNP** | **Effect Allele** | **Other Allele** | **β** | **SE** | **P-Value** | **F** |
| --- | --- | --- | --- | --- | --- | --- |
| rs1054402 | T | C | 0.1325 | 0.0269 | 8.201E-07 | 24.249 |
| rs11627423 | A | C | 0.1178 | 0.0246 | 1.65E-06 | 22.918 |
| rs11869294 | C | G | -0.2286 | 0.047 | 1.128E-06 | 23.644 |
| rs187166731 | T | C | -0.2424 | 0.0504 | 1.547E-06 | 23.119 |
| rs3876037 | A | G | 0.1234 | 0.027 | 4.733E-06 | 20.877 |
| rs4441609 | T | C | 0.1056 | 0.0231 | 4.746E-06 | 20.887 |
| rs61335305 | A | C | 0.4315 | 0.0904 | 1.812E-06 | 22.771 |
| rs6699436 | A | G | -0.1858 | 0.0404 | 4.365E-06 | 21.139 |

**Supplementary Table 11** Characteristics of the single nucleotide polymorphisms (SNP) used as instrumental variables for interleukin-2 (IL-2)

| **SNP** | **Effect Allele** | **Other Allele** | **β** | **SE** | **P-Value** | **F** |
| --- | --- | --- | --- | --- | --- | --- |
| rs13412535 | A | G | 0.174 | 0.0331 | 1.447E-07 | 27.618 |
| rs16836080 | A | G | 0.1158 | 0.0253 | 4.841E-06 | 20.938 |
| rs170117 | T | C | -0.1637 | 0.0347 | 2.442E-06 | 22.243 |
| rs4634519 | A | G | -0.1249 | 0.0268 | 3.18E-06 | 21.707 |
| rs61335305 | A | C | 0.4439 | 0.0913 | 1.157E-06 | 23.626 |
| rs62124990 | T | G | -0.7013 | 0.149 | 2.502E-06 | 22.141 |
| rs7615304 | A | G | -0.1139 | 0.024 | 2.161E-06 | 22.510 |

**Supplementary Table 12** Characteristics of the single nucleotide polymorphisms (SNP) used as instrumental variables for interleukin-2 receptor alpha subunit (IL-2rα)

| **SNP** | **Effect Allele** | **Other Allele** | **β** | **SE** | **P-Value** | **F** |
| --- | --- | --- | --- | --- | --- | --- |
| rs11241559 | T | G | -0.124 | 0.0264 | 2.75E-06 | 22.050 |
| rs117244812 | A | G | -0.7187 | 0.1493 | 1.474E-06 | 23.160 |
| rs12722497 | A | C | 0.6287 | 0.0482 | 7.978E-39 | 170.043 |
| rs12799226 | T | C | -0.1285 | 0.0277 | 3.561E-06 | 21.509 |
| rs185231391 | T | C | 0.8568 | 0.1803 | 2.004E-06 | 22.570 |
| rs28441585 | A | T | 0.1269 | 0.0271 | 2.934E-06 | 21.915 |
| rs4733117 | A | C | 0.1439 | 0.0291 | 7.912E-07 | 24.440 |
| rs759244 | A | T | -0.1094 | 0.0238 | 4.257E-06 | 21.118 |

**Supplementary Table 13** Characteristics of the single nucleotide polymorphisms (SNP) used as instrumental variables for interleukin-4 (IL-4)

| **SNP** | **Effect Allele** | **Other Allele** | **β** | **SE** | **P-Value** | **F** |
| --- | --- | --- | --- | --- | --- | --- |
| rs10512267 | T | C | -0.0824 | 0.016 | 2.734E-07 | 26.516 |
| rs116705532 | T | G | -0.4675 | 0.0978 | 1.727E-06 | 22.844 |
| rs117146485 | T | C | -0.2856 | 0.0625 | 4.945E-06 | 20.876 |
| rs12238729 | T | C | 0.5271 | 0.1096 | 1.505E-06 | 23.124 |
| rs13106889 | A | T | -0.1186 | 0.0224 | 1.215E-07 | 28.027 |
| rs17713451 | A | G | 0.1255 | 0.0252 | 6.41E-07 | 24.796 |
| rs2073438 | A | G | 0.0847 | 0.0183 | 3.725E-06 | 21.417 |
| rs6765768 | A | G | 0.0796 | 0.0167 | 1.853E-06 | 22.714 |
| rs6969391 | T | C | 0.0767 | 0.0166 | 3.587E-06 | 21.344 |
| rs73023729 | A | G | -0.1796 | 0.0365 | 8.561E-07 | 24.206 |
| rs7613691 | A | G | 0.1787 | 0.0382 | 2.962E-06 | 21.878 |
| rs9508291 | T | C | -0.168 | 0.0358 | 2.669E-06 | 22.016 |
| rs9941733 | A | G | 0.1156 | 0.0229 | 4.331E-07 | 25.476 |

**Supplementary Table 14** Characteristics of the single nucleotide polymorphisms (SNP) used as instrumental variables for interleukin-5 (IL-5)

| **SNP** | **Effect Allele** | **Other Allele** | **β** | **SE** | **P-Value** | **F** |
| --- | --- | --- | --- | --- | --- | --- |
| rs11680908 | A | G | 0.2593 | 0.0552 | 2.617E-06 | 22.053 |
| rs148634917 | A | G | -0.517 | 0.1087 | 1.974E-06 | 22.608 |
| rs28793375 | T | C | 0.1697 | 0.0362 | 2.746E-06 | 21.963 |
| rs6737109 | T | C | 0.1135 | 0.0246 | 3.806E-06 | 21.275 |
| rs72831687 | A | G | -0.5337 | 0.1104 | 1.324E-06 | 23.356 |
| rs73040130 | T | C | 0.2745 | 0.0525 | 1.709E-07 | 27.322 |
| rs74811276 | A | G | 0.217 | 0.0471 | 4.082E-06 | 21.214 |
| rs9472168 | A | G | 0.1568 | 0.0253 | 5.423E-10 | 38.388 |

**Supplementary Table 15** Characteristics of the single nucleotide polymorphisms (SNP) used as instrumental variables for interleukin-6 (IL-6)

| **SNP** | **Effect Allele** | **Other Allele** | **β** | **SE** | **P-Value** | **F** |
| --- | --- | --- | --- | --- | --- | --- |
| rs10752777 | A | T | 0.1083 | 0.0235 | 4.171E-06 | 21.233 |
| rs10982213 | A | G | -0.0849 | 0.0176 | 1.353E-06 | 23.264 |
| rs113098456 | A | G | -0.1553 | 0.0339 | 4.641E-06 | 20.982 |
| rs1333040 | T | C | 0.0747 | 0.0157 | 1.993E-06 | 22.633 |
| rs13412535 | A | G | -0.1186 | 0.0214 | 3.141E-08 | 30.707 |
| rs2404476 | A | G | 0.0734 | 0.0156 | 2.684E-06 | 22.133 |
| rs4684700 | T | C | -0.0747 | 0.0162 | 3.912E-06 | 21.257 |
| rs72831623 | A | G | 0.197 | 0.0369 | 9.29E-08 | 28.495 |
| rs73273528 | T | C | 0.268 | 0.0553 | 1.25E-06 | 23.481 |
| rs75101555 | C | G | -0.3625 | 0.0781 | 3.439E-06 | 21.538 |
| rs76856708 | T | C | 0.336 | 0.0697 | 1.427E-06 | 23.233 |

**Supplementary Table 16** Characteristics of the single nucleotide polymorphisms (SNP) used as instrumental variables for interleukin-7 (IL-7)

| **SNP** | **Effect Allele** | **Other Allele** | **β** | **SE** | **P-Value** | **F** |
| --- | --- | --- | --- | --- | --- | --- |
| rs10196226 | A | G | 0.1538 | 0.0327 | 2.501E-06 | 22.108 |
| rs117509142 | T | C | -0.3213 | 0.0684 | 2.599E-06 | 22.052 |
| rs141425475 | T | C | -0.4801 | 0.1018 | 2.39E-06 | 22.229 |
| rs17091524 | T | C | 0.5092 | 0.1015 | 5.244E-07 | 25.153 |
| rs1958987 | T | C | 0.1261 | 0.0263 | 1.604E-06 | 22.976 |
| rs218247 | C | G | -0.1343 | 0.0285 | 2.419E-06 | 22.193 |
| rs28793375 | T | C | 0.1644 | 0.036 | 4.866E-06 | 20.842 |
| rs62006410 | T | C | -0.1492 | 0.0302 | 7.588E-07 | 24.393 |
| rs6921438 | A | G | -0.3204 | 0.0246 | 8.707E-39 | 169.536 |
| rs77981494 | T | C | -0.5201 | 0.1055 | 8.225E-07 | 24.289 |
| rs78346957 | A | G | 0.4632 | 0.1008 | 0.0000043 | 21.104 |

**Supplementary Table 17** Characteristics of the single nucleotide polymorphisms (SNP) used as instrumental variables for interleukin-8 (IL-8)

| **SNP** | **Effect Allele** | **Other Allele** | **β** | **SE** | **P-Value** | **F** |
| --- | --- | --- | --- | --- | --- | --- |
| rs116726256 | T | C | -0.2247 | 0.0489 | 4.261E-06 | 21.103 |
| rs12075 | A | G | 0.1148 | 0.0235 | 9.969E-07 | 23.851 |
| rs12438669 | A | C | -0.1182 | 0.0252 | 2.597E-06 | 21.988 |
| rs141926526 | A | C | -0.6221 | 0.1308 | 1.96E-06 | 22.608 |
| rs183628733 | T | C | 0.6547 | 0.1417 | 3.821E-06 | 21.335 |
| rs2673604 | A | C | -0.118 | 0.0254 | 3.289E-06 | 21.570 |
| rs3786107 | A | G | 0.2463 | 0.0517 | 1.935E-06 | 22.683 |
| rs75840288 | A | C | 0.5125 | 0.1121 | 4.846E-06 | 20.890 |

**Supplementary Table 18** Characteristics of the single nucleotide polymorphisms (SNP) used as instrumental variables for interleukin-9 (IL-9)

| **SNP** | **Effect Allele** | **Other Allele** | **β** | **SE** | **P-Value** | **F** |
| --- | --- | --- | --- | --- | --- | --- |
| rs117807175 | C | G | -0.5225 | 0.1106 | 2.327E-06 | 22.306 |
| rs3736858 | C | G | -0.1351 | 0.0291 | 3.373E-06 | 21.542 |
| rs41294750 | T | C | 0.3442 | 0.0736 | 2.916E-06 | 21.859 |
| rs4880409 | T | C | -0.3552 | 0.0716 | 6.952E-07 | 24.597 |
| rs73443903 | A | C | 0.2162 | 0.046 | 2.569E-06 | 22.078 |
| rs76963786 | T | C | -0.2856 | 0.0556 | 2.775E-07 | 26.371 |

**Supplementary Table 19** Characteristics of the single nucleotide polymorphisms (SNP) used as instrumental variables for interleukin-10 (IL-10)

| **SNP** | **Effect Allele** | **Other Allele** | **β** | **SE** | **P-Value** | **F** |
| --- | --- | --- | --- | --- | --- | --- |
| rs10457128 | A | G | -0.0854 | 0.0172 | 6.956E-07 | 24.646 |
| rs10493718 | A | C | -0.1081 | 0.0222 | 1.068E-06 | 23.705 |
| rs10888839 | C | G | 0.1203 | 0.025 | 1.562E-06 | 23.150 |
| rs1530455 | T | C | 0.082 | 0.0174 | 2.527E-06 | 22.203 |
| rs2086656 | T | C | -0.08 | 0.017 | 2.589E-06 | 22.140 |
| rs282258 | T | C | 0.0993 | 0.0162 | 8.63E-10 | 37.563 |
| rs3002131 | C | G | 0.1191 | 0.026 | 4.592E-06 | 20.978 |
| rs3025021 | T | C | 0.0913 | 0.0194 | 2.609E-06 | 22.142 |
| rs383684 | A | G | 0.092 | 0.0197 | 3.168E-06 | 21.804 |
| rs41282660 | A | G | -0.1169 | 0.0254 | 4.234E-06 | 21.176 |
| rs6085948 | A | G | 0.0977 | 0.0202 | 1.28E-06 | 23.387 |
| rs6799107 | T | C | -0.095 | 0.0206 | 3.992E-06 | 21.262 |
| rs6921438 | A | G | -0.2876 | 0.0166 | 1.381E-67 | 300.088 |
| rs7088799 | T | G | -0.0815 | 0.0166 | 9.352E-07 | 24.098 |

**Supplementary Table 20** Characteristics of the single nucleotide polymorphisms (SNP) used as instrumental variables for interleukin-12p70 (IL-12p70)

| **SNP** | **Effect Allele** | **Other Allele** | **β** | **SE** | **P-Value** | **F** |
| --- | --- | --- | --- | --- | --- | --- |
| rs10761731 | A | T | -0.0965 | 0.0161 | 2.119E-09 | 35.917 |
| rs13209117 | A | G | 0.0981 | 0.0186 | 1.271E-07 | 27.810 |
| rs2123852 | T | C | 0.0942 | 0.0204 | 3.73E-06 | 21.318 |
| rs273702 | A | G | -0.127 | 0.027 | 2.522E-06 | 22.120 |
| rs282258 | T | C | 0.0726 | 0.0156 | 3.282E-06 | 21.653 |
| rs34291323 | T | C | 0.0954 | 0.0198 | 1.487E-06 | 23.209 |
| rs41282644 | A | G | 0.1401 | 0.0303 | 3.737E-06 | 21.374 |
| rs6532374 | T | C | -0.1033 | 0.0226 | 4.613E-06 | 20.887 |
| rs6921438 | A | G | -0.3784 | 0.016 | 5.78E-124 | 559.188 |
| rs6993770 | A | T | 0.0918 | 0.0188 | 1.06E-06 | 23.838 |
| rs71361173 | T | G | 0.1105 | 0.0238 | 3.572E-06 | 21.551 |
| rs72831623 | A | G | 0.1929 | 0.0367 | 1.509E-07 | 27.620 |
| rs782107 | A | G | 0.0765 | 0.0156 | 9.125E-07 | 24.042 |
| rs9472183 | A | G | -0.1006 | 0.0157 | 1.384E-10 | 41.048 |

**Supplementary Table 21** Characteristics of the single nucleotide polymorphisms (SNP) used as instrumental variables for interleukin-13 (IL-13)

| **SNP** | **Effect Allele** | | **Other Allele** | | **β** | **SE** | **P-Value** | **F** |
| --- | --- | --- | --- | --- | --- | --- | --- | --- |
| rs10995615 | T | C | | -0.1591 | | 0.0341 | 3.118E-06 | 21.757 |
| rs117795020 | A | G | | -0.3584 | | 0.0716 | 5.479E-07 | 25.042 |
| rs12623722 | A | G | | -0.1189 | | 0.0257 | 3.614E-06 | 21.392 |
| rs138854806 | A | G | | -0.4204 | | 0.0839 | 5.449E-07 | 25.093 |
| rs139083458 | T | C | | 0.9995 | | 0.211 | 2.165E-06 | 22.426 |
| rs27949 | T | C | | -0.1144 | | 0.025 | 4.827E-06 | 20.928 |
| rs6799107 | T | C | | -0.1472 | | 0.0299 | 8.661E-07 | 24.223 |
| rs6921438 | A | G | | -0.4139 | | 0.0242 | 1.283E-65 | 292.360 |
| rs7073807 | T | C | | 0.1618 | | 0.0354 | 4.77E-06 | 20.879 |
| rs75383097 | C | G | | -0.5369 | | 0.116 | 3.702E-06 | 21.411 |
| rs76339001 | A | T | | -0.4375 | | 0.0886 | 7.915E-07 | 24.370 |
| rs77955971 | A | C | | 0.4408 | | 0.0868 | 3.756E-07 | 25.775 |

**Supplementary Table 22** Characteristics of the single nucleotide polymorphisms (SNP) used as instrumental variables for interleukin-16 (IL-16)

| **SNP** | **Effect Allele** | **Other Allele** | **β** | **SE** | **P-Value** | **F** |
| --- | --- | --- | --- | --- | --- | --- |
| rs116135478 | A | G | 0.8296 | 0.1637 | 4.05E-07 | 25.668 |
| rs117217798 | T | C | -0.2064 | 0.044 | 2.772E-06 | 21.992 |
| rs117916513 | A | G | -0.4713 | 0.0982 | 1.605E-06 | 23.021 |
| rs1255143 | T | C | 0.1387 | 0.0241 | 8.529E-09 | 33.103 |
| rs144691581 | A | G | 0.4929 | 0.0958 | 2.668E-07 | 26.457 |
| rs1801020 | A | G | 0.1678 | 0.0271 | 5.628E-10 | 38.318 |
| rs4253283 | T | C | 0.1506 | 0.026 | 7.215E-09 | 33.532 |
| rs4778636 | A | G | -0.7286 | 0.063 | 6.208E-31 | 133.675 |
| rs9706053 | T | C | 0.4412 | 0.0928 | 1.976E-06 | 22.590 |

**Supplementary Table 23** Characteristics of the single nucleotide polymorphisms (SNP) used as instrumental variables for interleukin-17 (IL-17)

| **SNP** | **Effect Allele** | **Other Allele** | **β** | **SE** | **P-Value** | **F** |
| --- | --- | --- | --- | --- | --- | --- |
| rs117556572 | T | C | -0.5256 | 0.1097 | 1.66E-06 | 22.950 |
| rs12735700 | T | G | -0.0943 | 0.0206 | 4.5E-06 | 20.950 |
| rs149738638 | T | C | -0.1553 | 0.0337 | 4.106E-06 | 21.231 |
| rs17282552 | T | C | -0.2026 | 0.0403 | 4.876E-07 | 25.267 |
| rs3804753 | A | G | 0.0943 | 0.0166 | 1.376E-08 | 32.262 |
| rs61990749 | C | G | 0.1124 | 0.0226 | 6.569E-07 | 24.729 |
| rs78296352 | T | G | 0.2949 | 0.0645 | 4.809E-06 | 20.899 |
| rs78629931 | T | C | 0.236 | 0.0471 | 5.462E-07 | 25.100 |

**Supplementary Table 24** Characteristics of the single nucleotide polymorphisms (SNP) used as instrumental variables for interleukin-18 (IL-18)

| **SNP** | **Effect Allele** | **Other Allele** | **β** | **SE** | **P-Value** | **F** |
| --- | --- | --- | --- | --- | --- | --- |
| rs10414578 | T | C | -0.1817 | 0.0347 | 1.643E-07 | 27.404 |
| rs116383510 | A | C | -0.5412 | 0.1052 | 2.699E-07 | 26.451 |
| rs117266781 | T | C | 0.7051 | 0.1436 | 9.176E-07 | 24.097 |
| rs12420140 | A | G | -0.2479 | 0.0261 | 1.949E-21 | 90.165 |
| rs143370787 | C | G | -0.3447 | 0.066 | 1.749E-07 | 27.262 |
| rs17229943 | A | C | -0.3076 | 0.0463 | 3.062E-11 | 44.114 |
| rs1979967 | T | C | 0.14 | 0.0285 | 8.719E-07 | 24.117 |
| rs385076 | T | C | -0.2472 | 0.0247 | 1.559E-23 | 100.107 |
| rs4482818 | A | G | 0.1233 | 0.0243 | 4.108E-07 | 25.732 |
| rs610473 | A | G | 0.1274 | 0.0242 | 1.433E-07 | 27.699 |
| rs78623212 | T | C | 0.8322 | 0.1676 | 6.82E-07 | 24.642 |
| rs78716465 | A | G | 0.3173 | 0.0679 | 2.981E-06 | 21.825 |

(Rs116383510 and rs385076 were found to have horizontal pleiotropy by MR-PRESSO of IL-18 on Kidney Stone, so they were removed.）

**Supplementary Table 25** Characteristics of the single nucleotide polymorphisms (SNP) used as instrumental variables for interferon gamma-induced protein 10 (IP-10)

| **SNP** | **Effect Allele** | **Other Allele** | **β** | **SE** | **P-Value** | **F** |
| --- | --- | --- | --- | --- | --- | --- |
| rs113831257 | A | G | 0.3639 | 0.0641 | 1.388E-08 | 32.212 |
| rs143799975 | A | G | -0.7551 | 0.1638 | 4.012E-06 | 21.240 |
| rs34383175 | T | C | -0.3196 | 0.0653 | 9.904E-07 | 23.942 |
| rs4862111 | T | C | 0.1448 | 0.0317 | 4.844E-06 | 20.854 |
| rs6707974 | A | G | 0.1574 | 0.0337 | 3.027E-06 | 21.803 |
| rs75970138 | A | G | -0.4845 | 0.1037 | 2.994E-06 | 21.817 |
| rs7645625 | T | G | -0.1116 | 0.0236 | 2.192E-06 | 22.350 |
| rs79848609 | A | C | 0.2514 | 0.0535 | 2.637E-06 | 22.069 |
| rs8112909 | A | G | -0.139 | 0.0297 | 2.956E-06 | 21.892 |

**Supplementary Table 26** Characteristics of the single nucleotide polymorphisms (SNP) used as instrumental variables for macrophage colony-stimulating factor (M-CSF)

| **SNP** | **Effect Allele** | **Other Allele** | **β** | **SE** | **P-Value** | **F** |
| --- | --- | --- | --- | --- | --- | --- |
| rs116274860 | T | G | 0.8262 | 0.1739 | 2.029E-06 | 22.554 |
| rs117867915 | T | C | 0.5224 | 0.1096 | 1.874E-06 | 22.701 |
| rs11963606 | C | G | -0.5353 | 0.117 | 4.731E-06 | 20.916 |
| rs12962919 | T | C | 0.3025 | 0.0659 | 4.394E-06 | 21.054 |
| rs34089869 | T | C | 0.2194 | 0.0462 | 2.078E-06 | 22.534 |
| rs4269021 | C | G | -0.2459 | 0.0504 | 1.051E-06 | 23.785 |
| rs56367447 | T | C | -0.4878 | 0.0876 | 2.569E-08 | 30.983 |
| rs62294910 | A | G | 0.3472 | 0.0687 | 4.378E-07 | 25.521 |
| rs72723242 | T | G | -0.4969 | 0.1083 | 4.434E-06 | 21.035 |
| rs78296352 | T | G | 0.522 | 0.111 | 2.578E-06 | 22.098 |
| rs9387100 | T | C | -0.135 | 0.029 | 3.341E-06 | 21.653 |

**Supplementary Table 27** Characteristics of the single nucleotide polymorphisms (SNP) used as instrumental variables for monocyte chemotactic protein-1 (MCP-1)

| **SNP** | **Effect Allele** | **Other Allele** | **β** | **SE** | **P-Value** | **F** |
| --- | --- | --- | --- | --- | --- | --- |
| rs10744620 | T | C | 0.0783 | 0.0161 | 1.118E-06 | 23.647 |
| rs111995966 | T | G | 0.1428 | 0.0309 | 3.788E-06 | 21.352 |
| rs12073356 | A | G | -0.1436 | 0.031 | 3.489E-06 | 21.453 |
| rs12075 | A | G | 0.2186 | 0.0154 | 1.361E-45 | 201.444 |
| rs143815843 | A | G | -0.2049 | 0.0447 | 4.609E-06 | 21.007 |
| rs146522229 | T | C | -0.5942 | 0.1161 | 3.093E-07 | 26.188 |
| rs2036297 | A | G | 0.1182 | 0.016 | 1.302E-13 | 54.562 |
| rs2288370 | T | C | -0.1036 | 0.0162 | 1.555E-10 | 40.887 |
| rs56212190 | T | C | 0.1799 | 0.0372 | 1.318E-06 | 23.381 |
| rs7033586 | A | G | -0.22 | 0.0467 | 2.426E-06 | 22.187 |
| rs7197349 | A | G | 0.0971 | 0.0206 | 2.399E-06 | 22.213 |
| rs7517040 | A | G | -0.097 | 0.019 | 3.409E-07 | 26.057 |
| rs7632755 | A | G | 0.2984 | 0.0315 | 2.791E-21 | 89.717 |
| rs9317045 | A | C | 0.1157 | 0.0235 | 8.425E-07 | 24.234 |

**Supplementary Table 28** Characteristics of the single nucleotide polymorphisms (SNP) used as instrumental variables for monocyte specific chemokine 3 (MCP-3)

| **SNP** | **Effect Allele** | **Other Allele** | **β** | **SE** | **P-Value** | **F** |
| --- | --- | --- | --- | --- | --- | --- |
| rs117286643 | A | G | 0.6934 | 0.1474 | 2.542E-06 | 22.089 |
| rs2838065 | A | G | -0.221 | 0.0479 | 3.924E-06 | 21.248 |
| rs28394764 | A | T | 0.597 | 0.1282 | 3.194E-06 | 21.646 |
| rs3129806 | T | C | -0.1975 | 0.0433 | 4.978E-06 | 20.767 |
| rs62492260 | T | G | -0.2802 | 0.0578 | 1.229E-06 | 23.458 |

**Supplementary Table 29** Characteristics of the single nucleotide polymorphisms (SNP) used as instrumental variables for macrophage migration inhibitory factor (MIF)

| **SNP** | **Effect Allele** | **Other Allele** | **β** | **SE** | **P-Value** | **F** |
| --- | --- | --- | --- | --- | --- | --- |
| rs113218956 | A | G | -0.8789 | 0.1876 | 2.815E-06 | 21.937 |
| rs11551183 | C | G | 0.3666 | 0.0795 | 3.999E-06 | 21.252 |
| rs12594190 | A | G | 0.1321 | 0.0266 | 6.85E-06 | 24.649 |
| rs13142904 | T | C | -0.2232 | 0.0425 | 1.468E-07 | 27.565 |
| rs141009259 | T | C | -0.6194 | 0.1285 | 1.444E-06 | 23.221 |
| rs2294689 | C | G | -0.1338 | 0.0287 | 3.043E-06 | 21.722 |
| rs2330634 | C | G | 0.1549 | 0.0249 | 4.571E-10 | 38.677 |
| rs35890933 | T | G | 0.1676 | 0.0365 | 4.458E-06 | 21.073 |
| rs3814097 | A | G | -0.1163 | 0.0251 | 3.548E-06 | 21.457 |
| rs78098071 | T | C | -0.4583 | 0.0915 | 5.509E-07 | 25.073 |

**Supplementary Table 30** Characteristics of the single nucleotide polymorphisms (SNP) used as instrumental variables for monokine induced by interferon-gamma (MIG)

| **SNP** | **Effect Allele** | **Other Allele** | **β** | **SE** | **P-Value** | **F** |
| --- | --- | --- | --- | --- | --- | --- |
| rs111607343 | A | G | -0.5235 | 0.1119 | 2.928E-06 | 21.875 |
| rs11177248 | A | G | 0.3157 | 0.0667 | 2.222E-06 | 22.391 |
| rs112861654 | A | G | -0.2682 | 0.0527 | 3.639E-07 | 25.88588474 |
| rs117831247 | T | C | -0.8819 | 0.173 | 3.445E-07 | 25.972 |
| rs139010077 | T | C | 0.4337 | 0.0943 | 4.193E-06 | 21.141 |
| rs1796086 | T | C | -0.2172 | 0.04 | 5.623E-08 | 29.469 |
| rs55876513 | T | G | 0.1638 | 0.0254 | 1.048E-10 | 41.565 |
| rs62562991 | A | G | 0.6239 | 0.1259 | 7.237E-07 | 24.544 |
| rs6679677 | A | C | 0.1628 | 0.0327 | 6.514E-07 | 24.773 |
| rs77086208 | T | C | 0.327 | 0.0694 | 2.501E-06 | 22.189 |
| rs816960 | T | C | -0.1179 | 0.0242 | 1.15E-06 | 23.723 |

**Supplementary Table 31** Characteristics of the single nucleotide polymorphisms (SNP) used as instrumental variables for macrophage inflammatory protein 1 alpha (MIP-1α)

| **SNP** | **Effect Allele** | **Other Allele** | **β** | **SE** | **P-Value** | **F** |
| --- | --- | --- | --- | --- | --- | --- |
| rs12690897 | A | G | 0.1215 | 0.026 | 3.073E-06 | 21.825 |
| rs184154340 | A | G | 0.3251 | 0.0689 | 2.399E-06 | 22.251 |
| rs57786342 | A | G | 0.139 | 0.0283 | 8.909E-07 | 24.111 |
| rs60198979 | A | G | -0.2154 | 0.0455 | 2.215E-06 | 22.399 |

**Supplementary Table 32** Characteristics of the single nucleotide polymorphisms (SNP) used as instrumental variables for macrophage inflammatory protein 1 beta (MIP-1**β**)

| **SNP** | **Effect Allele** | **Other Allele** | **β** | **SE** | **P-Value** | **F** |
| --- | --- | --- | --- | --- | --- | --- |
| rs113010081 | T | C | -0.5799 | 0.0236 | 1.57E-133 | 603.639 |
| rs113877493 | T | C | -0.607 | 0.0217 | 3.67E-172 | 782.264 |
| rs116237296 | A | G | 0.5284 | 0.1115 | 2.153E-06 | 22.453 |
| rs117453826 | A | G | -0.5907 | 0.0591 | 1.53E-23 | 99.874 |
| rs117657747 | A | G | 0.2089 | 0.0453 | 4.013E-06 | 21.261 |
| rs141102180 | T | G | 0.3298 | 0.0392 | 3.751E-17 | 70.766 |
| rs1437220 | T | C | 0.1437 | 0.0315 | 4.916E-06 | 20.806 |
| rs1564708 | T | C | -0.1697 | 0.0187 | 1.294E-19 | 82.333 |
| rs17138331 | A | G | -0.1434 | 0.0295 | 1.125E-06 | 23.624 |
| rs2411161 | T | C | 0.1719 | 0.0365 | 2.547E-06 | 22.175 |
| rs281748 | C | G | -0.0794 | 0.0171 | 3.277E-06 | 21.555 |
| rs3760440 | A | G | 0.1242 | 0.0162 | 1.734E-14 | 58.764 |
| rs5743614 | T | C | 0.1115 | 0.0232 | 1.569E-06 | 23.092 |
| rs6908843 | A | G | 0.0997 | 0.0209 | 1.779E-06 | 22.751 |
| rs72791296 | T | C | 0.2364 | 0.0466 | 3.968E-07 | 25.729 |
| rs72799710 | T | C | -0.1037 | 0.0217 | 1.792E-06 | 22.831 |
| rs76356863 | A | T | -0.3456 | 0.0667 | 2.219E-07 | 26.840 |
| rs76582507 | A | G | 0.3259 | 0.0676 | 1.421E-06 | 23.236 |
| rs76776296 | A | G | 0.313 | 0.0598 | 1.632E-07 | 27.389 |
| rs79068918 | C | G | 0.2674 | 0.0271 | 5.537E-23 | 97.337 |

**Supplementary Table 33** Characteristics of the single nucleotide polymorphisms (SNP) used as instrumental variables for platelet derived growth factor BB (PDGF-BB)

| **SNP** | **Effect Allele** | **Other Allele** | **β** | **SE** | **P-Value** | **F** |
| --- | --- | --- | --- | --- | --- | --- |
| rs11247305 | C | G | -0.1687 | 0.0364 | 3.468E-06 | 21.475 |
| rs116445074 | T | G | 0.2869 | 0.0587 | 1.017E-06 | 23.883 |
| rs11766649 | A | G | 0.0902 | 0.0196 | 3.964E-06 | 21.174 |
| rs12289510 | A | G | -0.0772 | 0.0158 | 1.001E-06 | 23.868 |
| rs13037046 | A | T | -0.0948 | 0.0206 | 3.958E-06 | 21.173 |
| rs13412535 | A | G | 0.3317 | 0.0214 | 2.893E-54 | 240.192 |
| rs2324229 | T | C | 0.0884 | 0.0161 | 4.016E-08 | 30.140 |
| rs35859699 | A | G | -0.3854 | 0.0838 | 4.223E-06 | 21.146 |
| rs4965869 | T | C | 0.1843 | 0.0181 | 2.22E-24 | 103.655 |
| rs55680718 | T | C | -0.1359 | 0.0245 | 2.956E-08 | 30.761 |
| rs72777070 | T | G | -0.1048 | 0.02 | 1.557E-07 | 27.451 |
| rs73162807 | A | C | -0.2313 | 0.0499 | 3.548E-06 | 21.481 |
| rs9936075 | A | G | -0.0767 | 0.0163 | 2.675E-06 | 22.137 |
| rs9941733 | A | G | 0.1165 | 0.0227 | 3.018E-07 | 26.333 |

**Supplementary Table 34** Characteristics of the single nucleotide polymorphisms (SNP) used as instrumental variables for regulated on activation, normal T cell expressed and secreted (RANTES)

| **SNP** | **Effect Allele** | **Other Allele** | **β** | **SE** | **P-Value** | **F** |
| --- | --- | --- | --- | --- | --- | --- |
| rs112072646 | A | G | 0.4209 | 0.0859 | 9.617E-07 | 23.995 |
| rs147509526 | T | C | -0.3558 | 0.0715 | 6.567E-07 | 24.748 |
| rs2251660 | A | C | 0.1831 | 0.0356 | 2.691E-07 | 26.438 |
| rs2731672 | T | C | -0.1242 | 0.0272 | 4.827E-06 | 20.838 |
| rs62438851 | A | G | -0.1904 | 0.0413 | 4.009E-06 | 21.241 |
| rs7000423 | T | C | -0.1314 | 0.0252 | 1.852E-07 | 27.173 |
| rs7170339 | C | G | -0.4283 | 0.0904 | 2.187E-06 | 22.434 |
| rs72793342 | A | G | -0.1505 | 0.0307 | 9.08E-07 | 24.018 |
| rs74472919 | T | C | 0.3547 | 0.06 | 3.353E-09 | 34.928 |
| rs9675798 | T | G | -0.2583 | 0.0552 | 2.888E-06 | 21.884 |

（Rs2731672 was found to have horizontal pleiotropy by MR-PRESSO of RANTES on Kidney Stone, so it was removed.）

**Supplementary Table 35** Characteristics of the single nucleotide polymorphisms (SNP) used as instrumental variables for stem cell factor (SCF)

| **SNP** | **Effect Allele** | **Other Allele** | **β** | **SE** | **P-Value** | **F** |
| --- | --- | --- | --- | --- | --- | --- |
| rs113127926 | A | C | 0.1974 | 0.0418 | 2.337E-06 | 22.297 |
| rs13412535 | A | G | -0.1065 | 0.0213 | 5.586E-07 | 24.994 |
| rs1557570 | T | G | 0.1172 | 0.0169 | 4.133E-12 | 48.082 |
| rs1568119 | T | C | -0.5946 | 0.1129 | 1.374E-07 | 27.731 |
| rs4841899 | T | C | -0.1002 | 0.0178 | 1.673E-08 | 31.681 |
| rs635634 | T | C | -0.1035 | 0.0191 | 5.705E-08 | 29.357 |
| rs7039247 | C | G | 0.079 | 0.0168 | 2.46E-06 | 22.107 |
| rs72678285 | A | T | 0.1062 | 0.0231 | 4.43E-06 | 21.131 |
| rs78369473 | T | C | -0.2256 | 0.0484 | 3.14E-06 | 21.721 |
| rs78666213 | T | G | -0.2845 | 0.0574 | 7.152E-07 | 24.560 |
| rs80271436 | A | G | -0.2393 | 0.0484 | 7.488E-07 | 24.440 |

**Supplementary Table 36** Characteristics of the single nucleotide polymorphisms (SNP) used as instrumental variables for stem cell growth factor beta (SCGF-β)

| **SNP** | **Effect Allele** | **Other Allele** | **β** | **SE** | **P-Value** | **F** |
| --- | --- | --- | --- | --- | --- | --- |
| rs112346514 | T | C | -0.3261 | 0.0703 | 3.543E-06 | 21.506 |
| rs1149926 | T | C | -0.3458 | 0.0749 | 3.917E-06 | 21.304 |
| rs116924815 | T | C | 0.6046 | 0.0737 | 2.251E-16 | 67.262 |
| rs117716477 | A | C | 0.8242 | 0.084 | 1.029E-22 | 96.222 |
| rs12480722 | T | C | 0.1654 | 0.0353 | 2.812E-06 | 21.943 |
| rs13287050 | A | T | -0.121 | 0.0263 | 4.118E-06 | 21.156 |
| rs139413256 | A | G | -0.5174 | 0.1076 | 1.532E-06 | 23.110 |
| rs143829871 | T | C | -0.1866 | 0.0399 | 2.852E-06 | 21.860 |
| rs149009264 | A | G | 0.4551 | 0.0985 | 3.793E-06 | 21.336 |
| rs150733161 | T | C | -0.5255 | 0.112 | 2.687E-06 | 22.003 |
| rs151194174 | A | G | 0.4536 | 0.0941 | 1.454E-06 | 23.224 |
| rs17876031 | A | G | -0.1496 | 0.0254 | 3.667E-09 | 34.671 |
| rs264157 | A | G | 0.1079 | 0.0233 | 3.685E-06 | 21.434 |
| rs34911860 | A | G | -0.3674 | 0.0787 | 3.002E-06 | 21.782 |
| rs4656185 | A | G | 0.2103 | 0.0254 | 1.287E-16 | 68.514 |
| rs77954165 | T | C | 0.2631 | 0.0562 | 2.867E-06 | 21.905 |
| rs7815967 | T | C | 0.1325 | 0.0288 | 4.371E-06 | 21.155 |
| rs78217154 | T | C | 0.3942 | 0.0861 | 4.722E-06 | 20.950 |

**Supplementary Table 37** Characteristics of the single nucleotide polymorphisms (SNP) used as instrumental variables for stromal cell-derived factor 1 alpha (SDF-1α)

| **SNP** | **Effect Allele** | **Other Allele** | **β** | **SE** | **P-Value** | **F** |
| --- | --- | --- | --- | --- | --- | --- |
| rs10013755 | A | T | 0.5188 | 0.0995 | 1.851E-07 | 27.180 |
| rs10474392 | A | G | 0.0934 | 0.0177 | 1.376E-07 | 27.838 |
| rs12141941 | T | C | -0.0881 | 0.0186 | 2.263E-06 | 22.429 |
| rs149893336 | A | G | -0.494 | 0.1082 | 4.93E-06 | 20.840 |
| rs1600396 | A | G | -0.0933 | 0.0204 | 4.939E-06 | 20.912 |
| rs3988298 | T | C | -0.1263 | 0.0266 | 2.124E-06 | 22.539 |
| rs62194947 | T | C | -0.0852 | 0.0185 | 4.269E-06 | 21.204 |
| rs78037609 | A | G | -0.6261 | 0.1334 | 2.666E-06 | 22.022 |
| rs78883416 | C | G | -0.0871 | 0.0182 | 1.755E-06 | 22.897 |

（Rs10013755 and rs3988298 were found to have horizontal pleiotropy by MR-PRESSO of SDF-1α on Calculus of Lower Urinary Tract, so they were removed.）

**Supplementary Table 38** Characteristics of the single nucleotide polymorphisms (SNP) used as instrumental variables for tumor necrosis factor alpha (TNF-α)

| **SNP** | **Effect Allele** | **Other Allele** | **β** | **SE** | **P-Value** | **F** |
| --- | --- | --- | --- | --- | --- | --- |
| rs10834997 | A | G | -0.123 | 0.0256 | 1.528E-06 | 23.072 |
| rs115669577 | A | G | 0.981 | 0.1994 | 8.625E-07 | 24.190 |
| rs7256693 | T | C | -0.1841 | 0.04 | 4.109E-06 | 21.171 |
| rs79105320 | A | G | 0.5573 | 0.1177 | 2.207E-06 | 22.407 |

**Supplementary Table 39** Characteristics of the single nucleotide polymorphisms (SNP) used as instrumental variables for tumor necrosis factor beta (TNF-**β**)

| **SNP** | **Effect Allele** | **Other Allele** | **β** | **SE** | **P-Value** | **F** |
| --- | --- | --- | --- | --- | --- | --- |
| rs10925040 | T | C | 0.1738 | 0.0372 | 2.929E-06 | 21.800 |
| rs75240021 | C | G | 0.3713 | 0.0772 | 1.489E-06 | 23.103 |
| rs753274 | T | C | -0.1725 | 0.037 | 3.143E-06 | 21.708 |
| rs7629875 | A | G | 0.3841 | 0.0774 | 6.897E-07 | 24.595 |
| rs78296352 | T | G | 1.2028 | 0.1366 | 1.276E-18 | 77.434 |

**Supplementary Table 40** Characteristics of the single nucleotide polymorphisms (SNP) used as instrumental variables for TNF-related apoptosis inducing ligand (TRAIL)

| **SNP** | **Effect Allele** | **Other Allele** | **β** | **SE** | **P-Value** | **F** |
| --- | --- | --- | --- | --- | --- | --- |
| rs138987090 | A | G | -0.7264 | 0.0749 | 2.974E-22 | 94.033 |
| rs17434886 | T | C | -0.0918 | 0.0199 | 4.195E-06 | 21.275 |
| rs193112415 | T | C | -1.0456 | 0.062 | 1.008E-63 | 284.343 |
| rs28431810 | C | G | -0.1216 | 0.0252 | 1.406E-06 | 23.279 |
| rs57396456 | T | C | -0.5641 | 0.0516 | 7.71E-28 | 119.483 |
| rs62093514 | T | C | 1.0459 | 0.0549 | 5.798E-81 | 362.852 |
| rs72899452 | T | C | 0.1223 | 0.0264 | 3.748E-06 | 21.456 |
| rs73039026 | A | C | -0.3098 | 0.0634 | 1.015E-06 | 23.871 |
| rs747324 | T | C | -0.0826 | 0.0178 | 3.338E-06 | 21.529 |
| rs74778900 | T | C | 0.5791 | 0.0531 | 9.901E-28 | 118.908 |
| rs75928541 | A | G | 0.2784 | 0.0591 | 2.442E-06 | 22.185 |
| rs79287178 | A | G | -0.4304 | 0.042 | 1.173E-24 | 104.988 |

**Supplementary Table 41** Characteristics of the single nucleotide polymorphisms (SNP) used as instrumental variables for vascular endothelial growth factor (VEGF)

| **SNP** | **Effect Allele** | **Other Allele** | **β** | **SE** | **P-Value** | **F** |
| --- | --- | --- | --- | --- | --- | --- |
| rs10411345 | C | G | -0.1041 | 0.0218 | 1.733E-06 | 22.796 |
| rs10761731 | A | T | -0.1146 | 0.0174 | 4.309E-11 | 43.366 |
| rs10934631 | T | C | -0.1132 | 0.0244 | 3.607E-06 | 21.518 |
| rs10967186 | T | C | 0.0899 | 0.0169 | 1.086E-07 | 28.289 |
| rs12456390 | T | C | -0.0818 | 0.0179 | 4.882E-06 | 20.878 |
| rs13209117 | A | G | 0.1253 | 0.02 | 3.697E-10 | 39.239 |
| rs143479231 | A | G | -0.2628 | 0.0489 | 7.902E-08 | 28.874 |
| rs3108686 | A | C | -0.7967 | 0.1702 | 2.861E-06 | 21.905 |
| rs4082730 | A | G | 0.2455 | 0.0533 | 4.116E-06 | 21.209 |
| rs6921438 | A | G | -0.4866 | 0.0174 | 4.11E-172 | 781.852 |
| rs7030781 | A | T | 0.1403 | 0.0172 | 3.454E-16 | 66.518 |
| rs73418461 | A | G | -0.2498 | 0.0521 | 1.61E-06 | 22.982 |
| rs73872715 | T | C | -0.6079 | 0.1299 | 2.864E-06 | 21.894 |
| rs8045833 | A | G | 0.103 | 0.0211 | 1.006E-06 | 23.823 |
| rs9472183 | A | G | -0.1264 | 0.017 | 9.541E-14 | 55.268 |

**Supplementary Table 42** Characteristics of the single nucleotide polymorphisms (SNP) used as instrumental variables for calculus of kidney and ureter (CALCUKIDUR)

| **SNP** | **Effect Allele** | **Other Allele** | **β** | **SE** | **P-Value** | **F** |
| --- | --- | --- | --- | --- | --- | --- |
| rs11750874 | C | A | -0.136072 | 0.0218797 | 5E-10 | 38.670 |
| rs147750540 | A | G | 0.294268 | 0.0286572 | 9.77E-25 | 105.421 |
| rs2079742 | C | T | -0.169637 | 0.0199165 | 1.63E-17 | 72.531 |
| rs219773 | A | G | -0.162294 | 0.0183378 | 8.73E-19 | 78.311 |
| rs2206271 | A | T | 0.0880942 | 0.0152902 | 8.34E-09 | 33.188 |
| rs4498196 | C | A | -0.0867456 | 0.0152922 | 1.41E-08 | 32.171 |
| rs57719175 | A | G | -0.0857595 | 0.0151043 | 1.36E-08 | 32.231 |
| rs6127099 | T | A | -0.151482 | 0.0169046 | 3.22E-19 | 80.283 |
| rs71606723 | T | A | 0.121761 | 0.0153748 | 2.38E-15 | 62.706 |
| rs925813 | C | T | -0.155524 | 0.0244633 | 2.05E-10 | 40.409 |
| rs9902482 | T | C | -0.0836725 | 0.0146671 | 1.17E-08 | 32.538 |

**Supplementary Table 43** Characteristics of the single nucleotide polymorphisms (SNP) used as instrumental variables for calculus of lower urinary tract (CALCULOWER)

| **SNP** | **Effect Allele** | **Other Allele** | **β** | **SE** | **P-Value** | **F** |
| --- | --- | --- | --- | --- | --- | --- |
| rs72646050 | A | G | -0.482119 | 0.104411 | 3.88E-06 | 21.291 |
| rs13097866 | A | T | -0.20366 | 0.0435526 | 2.92E-06 | 21.835 |
| rs62310034 | C | G | -0.382781 | 0.0837947 | 4.92E-06 | 20.838 |
| rs76483905 | T | C | -0.741001 | 0.161846 | 4.68E-06 | 20.932 |
| rs192666551 | A | T | -0.857005 | 0.171969 | 6.24E-07 | 24.800 |
| rs1547551 | C | G | -0.178148 | 0.0390097 | 4.95E-06 | 20.825 |
| rs4127501 | T | C | 0.200221 | 0.0401538 | 6.15E-07 | 24.828 |
| rs59023232 | T | C | 0.219654 | 0.0468041 | 2.69E-06 | 21.993 |
| rs1077219 | A | G | 0.403751 | 0.0768299 | 1.48E-07 | 27.577 |
